# Symbiotic Diversity of Sap-Feeding Auchenorrhyncha (Hemiptera) in the Upland Landscapes of Central Cardamom Mountains, Cambodia

**DOI:** 10.1007/s00248-026-02724-3

**Published:** 2026-02-28

**Authors:** Sophany Phauk, Sopha Sin, Olle Terenius

**Affiliations:** 1 Department of Cell and Molecular Biology, Microbiology, Uppsala University, Uppsala, Sweden; 2 Department of Biology, Faculty of Science, Royal University of Phnom Penh, Phnom Penh, Cambodia; 3 Centre for Biodiversity Conservation, Faculty of Science, Royal University of Phnom Penh, Phnom Penh, Cambodia

**Keywords:** Auchenorrhyncha, Symbionts, Cardamom mountains, Upland landscape, Microbiota, 16S rRNA

## Abstract

Auchenorrhyncha (Hemiptera) harbor diverse bacterial symbionts that play critical roles in host nutrition, adaptation and vector competence. However, how symbiotic communities vary across host species and ecological gradients in tropical montane ecosystems remains poorly understood. Here, we characterized the bacterial microbiota of eight auchenorrhynchan species collected from the upland landscapes of the Central Cardamom mountains, Cambodia, using 16S rRNA (V3-V4) amplicon sequencing. In total, 83 individuals representing eight species were analyzed. Across 188 amplicon sequence variants (ASVs), the obligate symbiont *Candidatus* (*Ca.*) Karelsulcia muelleri dominated all hosts, although its relative abundance varied substantially. While some species (*Anagonalia* sp., *Changwhania* sp. and *Mukaria* sp.) contained nearly exclusively *Karelsulcia*, others harbored diverse co-obligate (*Ca.* Zinderia) and secondary symbionts, including *Rickettsia*,* Wolbachia*,* Arsenophonus*,* Spiroplasma*,* Ca.* Symbiodolus, *Ca.* Lariskella, *Pectobacterium* and *Xylella*. Symbiont composition was highly host-specific, with *Clovia* sp. exhibiting the greatest diversity and *Hecalus* sp. dominated by *Rickettsia*. β-diversity analysis confirmed that host species explained most variation (87%), whereas topographic variables (exposure level and mountain locality) contributed little, although *Ca.* Symbiodolus in *Stirellus* sp2. was more frequent at high-exposure sites. Cross-correlation analysis revealed that only a restricted subset of bacterial taxa correlated significantly with plants families, suggesting selective ecological filtering. Notably, *Spiroplasma* was positively associated with sundew plants (Droseraceae), whereas *Pectobacterium* showed consistent negative correlations with multiple plant families. Overall, these findings demonstrate that microbiota in Auchenorrhyncha are shaped primarily by host species, with minor influences from environment and plant associations, highlighting the ecological and evolutionary drivers of symbiotic diversity in sap-feeding insects inhabiting tropical mountain landscapes.

**Supplementary Information:**

The online version contains supplementary material available at 10.1007/s00248-026-02724-3.

## Introduction

Auchenorrhyncha, a suborder within Hemiptera, encompasses a diverse group of sap-feeding insects, including cicadas, leafhoppers, treehoppers, spittlebugs, and planthoppers [1, 2, 3]. These insects maintain intricate relationships with microbial communities, particularly endosymbiotic bacteria, which are crucial for their survival and development [4, 5, 6]. Feeding primarily on nutrient-deficient plant sap, Auchenorrhyncha rely on these symbionts to supplement essential nutrients, such as amino acids and vitamins, that are lacking in their diet [7, 8, 9]. This mutualistic association has evolved over millions of years, leading to highly specialized and interdependent relationships between the host insects and their microbial partners [1, 5, 10]. Increasing evidence indicates that the microbial community assembly reflects the combined influence of deterministic processes, such as host filtering and environmental selection, and stochastic processes, including dispersal limitation and ecological drift [11, 12, 13].

One of the most prevalent endosymbionts in Auchenorrhyncha is *Candidatus (Ca.)* Karelsulcia muelleri (hereafter *Karelsulcia*), a bacterium from the Bacteroidetes phylum [14, 15, 16]. *Karelsulcia* is considered an obligate symbiont, present across various Auchenorrhyncha lineages [1, 4], and is primarily housed within specialized host cells known as bacteriocytes [8, 16]. This bacterium plays a vital role in synthesizing essential amino acids that the host cannot obtain from its sap-based diet. In many cases, *Karelsulcia* coexists with secondary (or co-obligate) symbionts, which complement its metabolic functions by providing additional nutrients or aiding in other physiological processes [4, 17, 18]. This co-symbiotic relationship highlights the co-evolutionary complexity of microbial communities within these insects. For instance, leafhoppers in the family Cicadellidae likewise depend on microbial symbionts, with primarily harboring *Karelsulcia* as their main endosymbiont [1, 4]. Additionally, they often possess co-obligate symbionts, such as *Candidatus* Nasuia deltocephalinicola (hereafter *Nasuia*), a Betaproteobacterium [19, 20]. *Nasuia* has an extremely reduced genome and together with *Karelsulcia*, provide a complete set of essential amino acids to the host [9, 19]. This dual symbiotic system is indicative of a long-term co-evolutionary relationship, enabling leafhoppers to thrive on their restrictive diets [1, 21].

Research on specific leafhopper species, such as *Macrosteles laevis*, has revealed insights into the stability and distribution of their microbial communities [22, 23]. Studies have shown limited variation in the composition of bacterial symbionts across different populations and over time, suggesting a stable and conserved symbiotic relationship [24]. This stability is crucial for the host’s fitness, as the consistent presence of essential symbionts ensures the continuous supply of necessary nutrients [9]. Moreover, the conservation of these microbial communities across various environments underscores their importance in the host’s biology and ecological success [25]. Another notable example is *Scaphoideus titanus*, a leafhopper species recognized as the primary vector of *Candidatus* Phytoplasma vitis, the causative agent of Flavescence dorée in grapevines [22, 26, 27]. Investigations into the bacterial communities of *S. titanus* across different European populations and life stages have identified *Karelsulcia* and *Candidatus* Cardinium as predominant symbionts present in all individuals. Interestingly, although some studies have reported that geographical variation can influence microbial composition accross the European populations of certain leafhoppers, these studies did not find any significant differences between nymphal and adult developmental stages [27].

Despite the well-established roles of primary (obligate) symbionts such as *Karelsulcia* and Nasuia, far less is known about how secondary symbionts vary across host species and ecological conditions. Obligate symbionts are typically highly conserved and tightly associated within host biology [1, 5], whereas secondary or facultative symbionts may exhibit greater variability driven by ecological, physiological, or environmental factors [8, 28]. Tropical mountain landscapes—such as the Central Cardamom Mountains—present strong ecological gradients, including variation in altitude, light exposure, habitat types, vegetation structure and micro-climate; yet the influence of these factors on Auchenorrhyncha-associated microbiota remains poorly understood. Although previous studies have showed that geographic and ecological factors can shape symbiont prevalence and community stability [4, 24, 27], evidence from tropical montane systems is still limited. Understanding whether topography and environmental gradients influence symbiont diversity and community structure is therefore essential for assessing the ecological resilience and adaptability of these insect-microbe associations in complex landscapes.

Furthermore, there is a notable scarcity of research on the symbionts of Auchenorrhyncha in Southeast Asia. Microbial community data for this insect group remain extremely limited in the region, with the exception of a recent report on rice-associated leafhoppers (Cicadellidae) from the Tonle Sap Lake floodplain [29]. In addition to host- and environment driven factors, interactions with local vegetation may further contribute to structure insect-associated microbiota. Because Auchenorrhyncha are sap-feeders that rely on diverse plants, differences in plant community composition across habitats may influence microbial persistence and acquisition, thereby shaping the composition of insect-associated microbial communities [24]. Plant traits such as phloem chemistry, defensive compounds, and ecological niches may directly or indirectly influence the prevalence and composition of microbial communities in insect hosts [26, 30]. Despite increasing recognition of plant-microbe-insect interactions, few studies have examined correlations between plant communities and the microbial assemblage of Auchenorrhyncha, leaving an important gap in understanding the ecological context of these symbioses.

In this study, we investigated symbiont communities associated with eight species of Auchenorrhyncha collected from the upland landscapes of the Central Cardamom Mountains in Cambodia. Specifically, we aimed (1) to characterize the diversity and composition of primary and secondary symbionts in different host species, (2) to evaluate the effect of topographic variation, including mountains and exposure levels, on insect-associated bacterial communities, and (3) to assess correlations between bacterial taxa and host-plant families across the study sites. We hypothesize that obligate symbiont will remain highly conserved across host species, whereas secondary symbionts will show greater variability and be influenced by environmental gradients and host-plant associations. Together, these objectives provide new insights into the stability, flexibility, and ecological drivers of symbiotic communities in sap-feeding insects from tropical montane ecosystems.

## Materials and Methods

### Study Site and Sample Collection

**Fig. 1:**
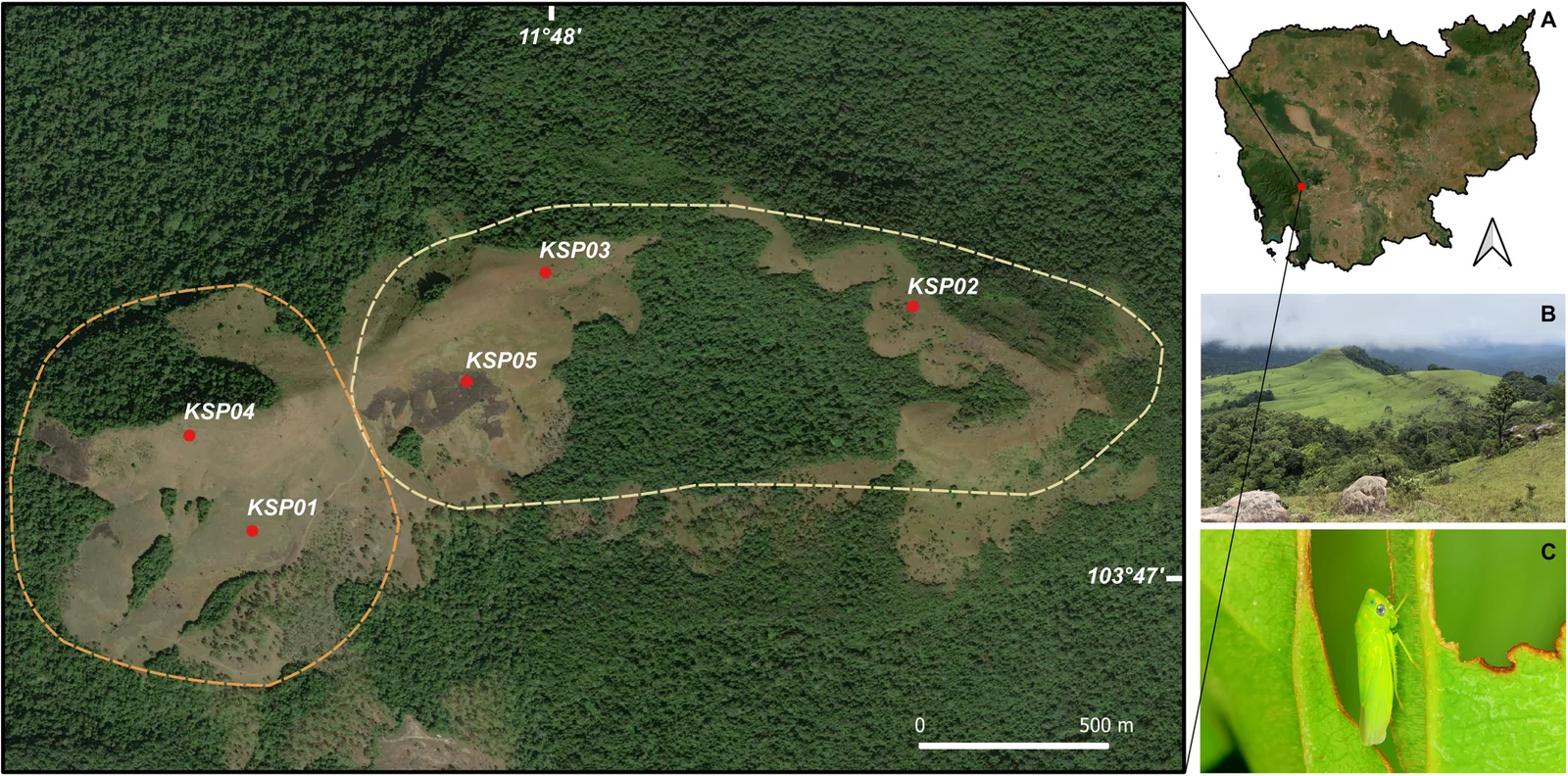
(**A**) Map of the study showing sampling sites within the Khnang Sampov (orange dashed line) and Khnang Phsar (yellow dashed line) upland landscapes in the Cardamom Mountains National Park. (**B**) Grazing habitat at the sampling sites. (**C**) A sap-feeding leafhopper (*Anagonalia* sp.)

The Khnang Phsar and Khnang Sampov upland landscapes are located within the Central Cardamom Mountains National Park, along the border between the Koh Kong and Kampong Speu provinces, Cambodia (11°46.731’N, 103°46.592’E, elv. 1,030 m. a.s.l). The research area has recently became a popular hiking destination for both local communities and visitors [31]. The region features a distinctive natural landscape characterized by open grasslands dominated by Poaceae, patches of pine forest at the southern part, and evergreen forest at the north (Fig. 1). The area experiences high humidity and a mean annual temperature of approximately 20 °C, ranging from 10 °C to 32 °C.

The survey was conducted in September 2020. Two exposure levels, representing high and low environmental conditions, were defined based on differences in altitude, sunlight intensity, wind exposure and habitat openness across five sampling sites (KS01, KS02, KS03, KS04 and KS05) (Table 1). Insects were collected using sweep-netting through vegetation within sampling site and were transferred with aspirators into 1.5 mL Eppendorf vials containing 95% ethanol. They were not directly observed feeding, ovipositing or completing their life cycle on specific plant species; therefore, plant data represent the surrounding vegetation community rather than confirmed host plants. All samples were deposited at the Cambodian Entomology Initiatives (CEI), Royal University of Phnom Penh (RUPP) in Cambodia, for sorting and identification into morphospecies. At each site, vegetation was surveyed using 1 × 1 m quadrats with three replications to document plant assemblages associated with the insect sampling plots. Plant specimens within each quadrat were collected and examined based on morphological characteristics following standard botanical identification procedures. All specimens were identified and assigned to their respective families by the second author and subsequently deposited in the National Herbarium of Cambodia at RUPP. For each quadrat, the total number of identifiable plants was calculated and from this the relative abundance was estimated in percent.

**Table 1:** Study site and topographical gradients of upland landscape, Cardamom Mountains National Park

No.	Site	Coordinates	Exposure*	Mountains	Altitude (m)
1	KPS01	11°47’0.28"N 103°46’23.61"E	Low	Khnang Sampov	915
2	KPS02	11°47’20.00"N 103°47’21.80"E	High	Khnang Phsar	1025
3	KPS03	11°47’23.10"N 103°46’48.43"E	High	Khnang Sampov	1000
4	KPS04	11°47’8.72"N 103°46’19.24"E	High	Khnang Phsar	971
5	KPS05	11°47’13.34"N 103°46’42.04"E	Low	Khnang Phsar	931

*Exposure level: defined as the degree of environmental influence at each site,* measured by altitude (low/high)*,* sunlight (shade/sun)*,* wind (sheltered/windy)*,* and habitat openness (closed/open)*

## Auchenorrhyncha Species

A total of 83 individuals of eight species of auchenorrhynchan insects were identified, comprising six Membracoidea (*Anagonalia* sp., *Changwhania* sp., *Hecalus* sp., *Mukaria* sp., *Stirellus* sp1. and *Stirellus* sp2.), one Fulgoroidea (*Symplanella* sp.) and one Cercopoidea (*Clovia* sp.) (Table S1). Morphological identifications were carried out by the first author and verified by Christopher H. Dietrich (Illinois Natural History Survey, INHS), and representative specimens were further examined using COI barcoding (primers LCO1490/HCO2198; Fig. 4 and S2). Species-level identification was attempted but was not possible for several taxa due to unresolved regional taxonomy, limited reference COI data and morphological similarity among closely related species; specimens were therefore conservatively assigned to genus level and treated as distinct morphospecies. Voucher specimens are deposited at the CEI and corresponding COI accession numbers are provided in Table S4. It is important to note that *Stirellus* sp1. and *Stirellus* sp2. analyzed in this study represent different morphospecies from those previously reported in Phauk et al., 2025 [28, 29].

## DNA Extraction and PCR Amplification

Prior to DNA extraction, insects were sterilized by sequential washing in 70% ethanol followed by rinsing in sterile distilled water to minimize external bacterial contamination. A total of eighty-three auchenorrhynchan samples (whole body), with sample sizes ranging from 6 to 13 individuals per species (Table 2) were then DNA extracted and purified by using QIAamp DNA Mini Kit Protocol (Qiagen) with addition of 20 mg mL^− 1^ of lysozyme enzyme (Table S1). The final elution DNA template of 125 µL per sample was used in the study. No extraction blank controls were included in this sequencing run, however, two mock microbial community standards (*Zymo*BIOMICs, Zymo Research, Irvine, CA, USA) were processed in parallel as positive controls to monitor amplification and sequencing performance (Figure S1).

PCR amplification of extracted DNA (*n* = 83) was performed by using a two-step method as described in [32], to generate barcoded bacterial 16S rDNA gene amplicons for sequencing. The bacterial 16S rDNA target regions (V3-V4) were amplified by using general bacterial primers 341F (5’-CCT ACG GGN GGC WGC AG-3’) and 805R (5’-GAC TAC HVG GGT ATC TAA TCC-3’) [33]. Per PCR reaction, 50–120 ng DNA was used as templates in the first step PCR. The first-step PCR program was performed by an initial denaturation at 95 °C for 5 min, followed by 25 cycles of [95 °C for 40 s, 53 °C for 40 s and 72 °C for 60 s], and a final elongation at 72 °C for 7 min. First step-PCRs were analyzed by using Gel Electrophoresis and quantified the DNA by Image Lab 6.0 software. PCR products were diluted in nuclease-free water to a concentration of 0.1-1 ng µL^− 1^ for the next step. In the second step PCR, 1 µL was used as a template from diluted PCR products and PCR was performed by adding 1 of 50 different barcoding primer pairs (Table S5). To be able to pool 50 samples per sequencing pool, different barcoding primer pairs were applied [34]. The second-step PCR program is using the same program as of the first step-PCR, but only for 10 cycles. The resulting PCR products were pooled, purified and eluted in 50 µL nuclease-free water. For all PCR reactions, Illustra PuReTaq-To-Go PCR Beads (GE Health Care) were used.

## High-throughput Sequencing and Bioinformatics

Amplicon sequencing using *MiSeq* technology was carried out at the SNP&SEQ Technology Platform, Science for Life Laboratory (SciLifeLab) at Uppsala University, Sweden (https://snpseq.medsci.uu.se/). Libraries were prepared with 5 ng of DNA per sample. Paired-end sequencing was conducted using 300 bp read lengths and v3 chemistry on the *MiSeq* system (Illumina), according to the manufacturer’s guidelines. To improve sequence quality, a PhiX phage library was included as a 10% spike-in during the sequencing run.

Raw .*fastq* files generated by the sequencing facility were processed at the Uppsala Multidisciplinary Center for Advanced Computational Science (UPPMAX), under project NAISS 2025/22–339, supported by the Swedish National Infrastructure for Computing (SNIC). Demultiplexing was performed with *Cutadapt* (v. 4.1) [35], targeting paired barcodes (*--pair-adapters*) under default parameters. PCR primers (341 F-805R) were removed, allowing up to 10% mismatches (one error for the 17-nt forward primer and two errors for the 21-nt reverse primer). Reads lacking detectable primers were discarded.

Quality-based trimming was then carried out with *TrimGalore* (v. 0.6.7) [36], using default settings. The trimmed reads were processed with *DADA2* (v. 1.26.0) in R (v. 4.2.0) [37], which included error modeling, Inference of amplicon sequence variants (ASVs) using pooled processing, and paired-end read merging. ASVs were filtered to retain only sequences of 350–470 nt in length, and chimeric were removed using *DADA2’s ‘removeBimeraDenovo’* function. Taxonomic classification of ASVs was performed using the *DECIPHER* package (v. 2.26.0) using the *IDTAXA* algorithm [38], aligning both strands against a modified *SILVA SSL* (r138) database [39].

The ASV count table, taxonomy table and ASV sequences list were generated. The ASV sequences were aligned with *MAFFT* (v. 7.508) [40], using default ‘*-auto’* settings. This alignment was then used to construct an unrooted phylogenetic tree with *IQ-TREE* (v. 2.2.0.3) [41], employing *ModelFinder Pro ‘MFP’* for model selection. All outputs—including the ASV counts, taxonomy, and phylogeny tree—were imported into the *phyloseq* package (v. 1.42.0) [42], for downstream analyses. Within *phyloseq*, sequences (ASVs) unclassified at the phylum level, or those assigned to *Chloroplast* or *Mitochondria*, were removed. The results were assembled in a *phyloseq* object and saved as an .*RData* file for further analyses.

## Microbial Analysis and Visualization

**Fig. 2:**
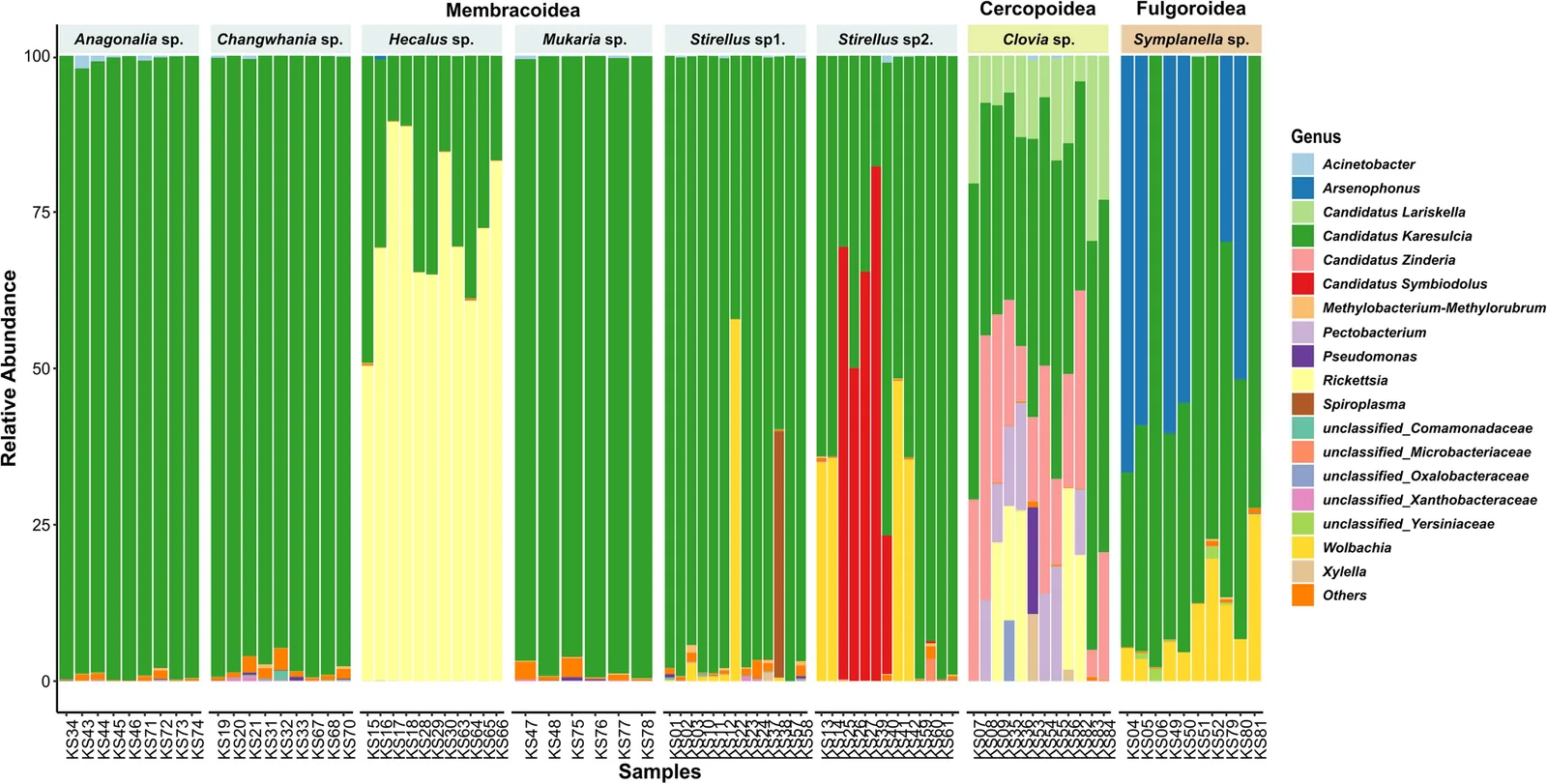
Bacterial communities associated with eight species of Auchenorrhyncha insects. Compositional bar plots showing the top eighteen bacterial genera detected across the eight auchenorrhynchan species

**Fig. 3:**
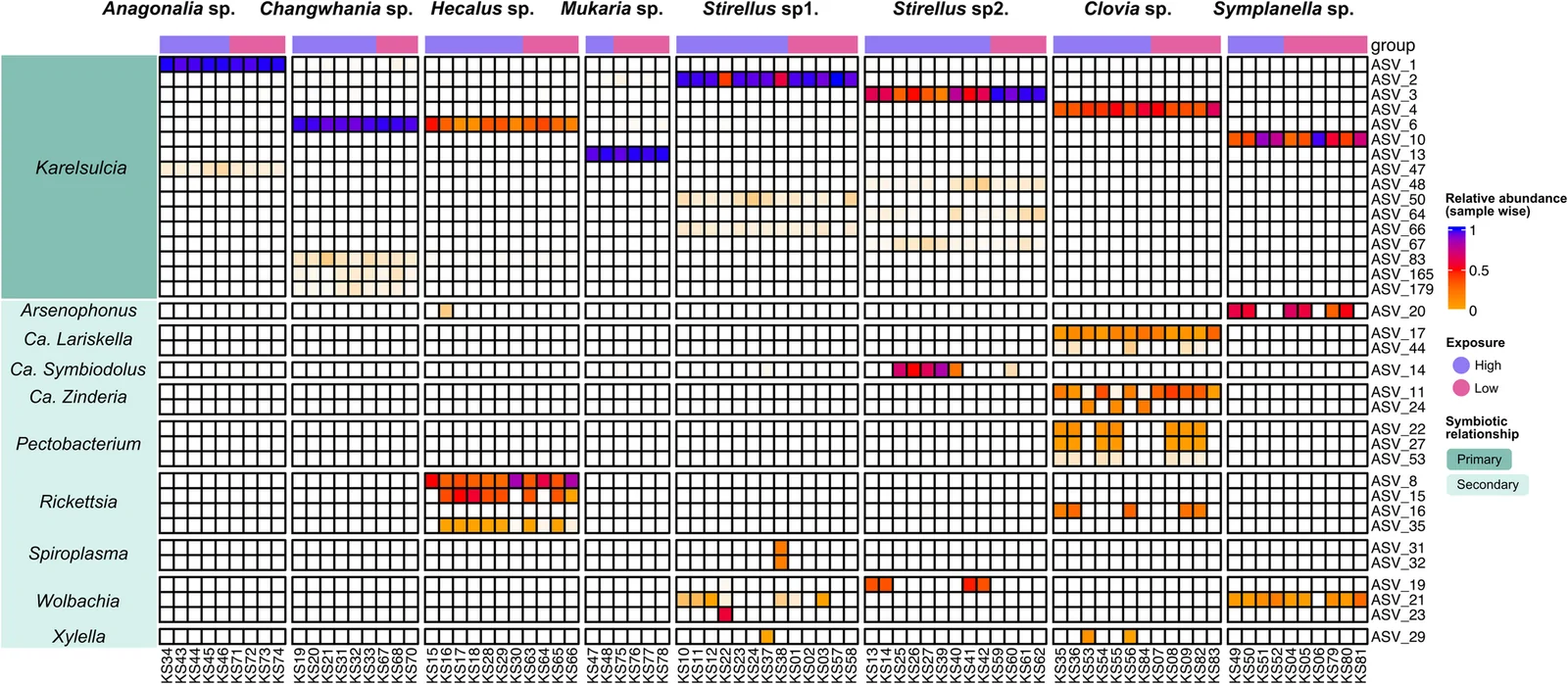
Heatmap of endosymbionts associated with Auchenorrhyncha hosts: The heatmap shows ASVs of primary/secondary symbionts for auchenorrhynchan insects, and their relative abundance (calculated sample-wise on the total number of reads per sample). To avoid the presence of singletons and low-abundance ASVs the lower limit of the scale has been set to 0.01 (1%) of relative abundance

**Fig. 4:**
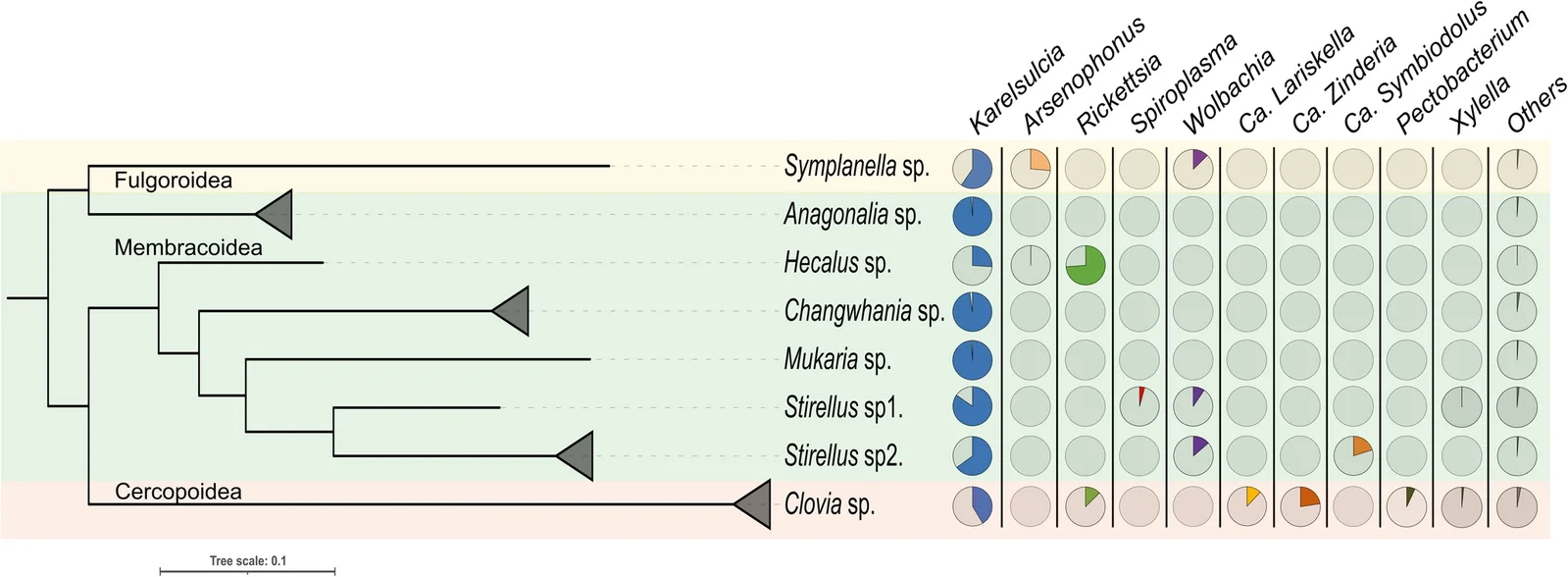
Auchenorrhynchan host-symbiont associations: the graph illustrates the phylogenetic relationships of auchenorrhynchan species and their associated symbiotic bacterial communities. On the left, a phylogenetic tree of host species is shown, based on the COI gene. Black triangles indicate cases when two or three samples of the sample species were collapsed. On the right, multiple pie-charts display the relative abundance of symbiotic bacteria associated with each host species

All downstream analyses were performed in R (v. 4.3.2). Amplicon sequence data were imported into a *phyloseq* object (v. 1.42.0) [42], for integrated microbiota analysis. To examine microbial variation across auchenorrhynchan insects, bacterial communities were examined at both the phylum and genus levels. Taxonomic composition (at the genus level) was visualized as stacked bar plots using with *ggplot2* package [43] (Fig. 2). Host-symbiont associations (Fig. 3) were conducted using the ComplexHeatmap package (v. 2.14.0) [44]. Phylogenetic relationships of host-symbiont associations were constructed using multiple sequence alignment performed with *MAFFT*, followed by maximum likelihood tree inference in *IQ-TREE* with *ModelFinder Pro ‘MFP’* for model selection and 1,000 ultrafast bootstrap replicates for branch support. The resulting phylogenies (Figs. 2A and 4) were visualized using the Interactive Tree of Life (iTOL, v. 7.2.1) web portal [45].

**Fig. 5:**
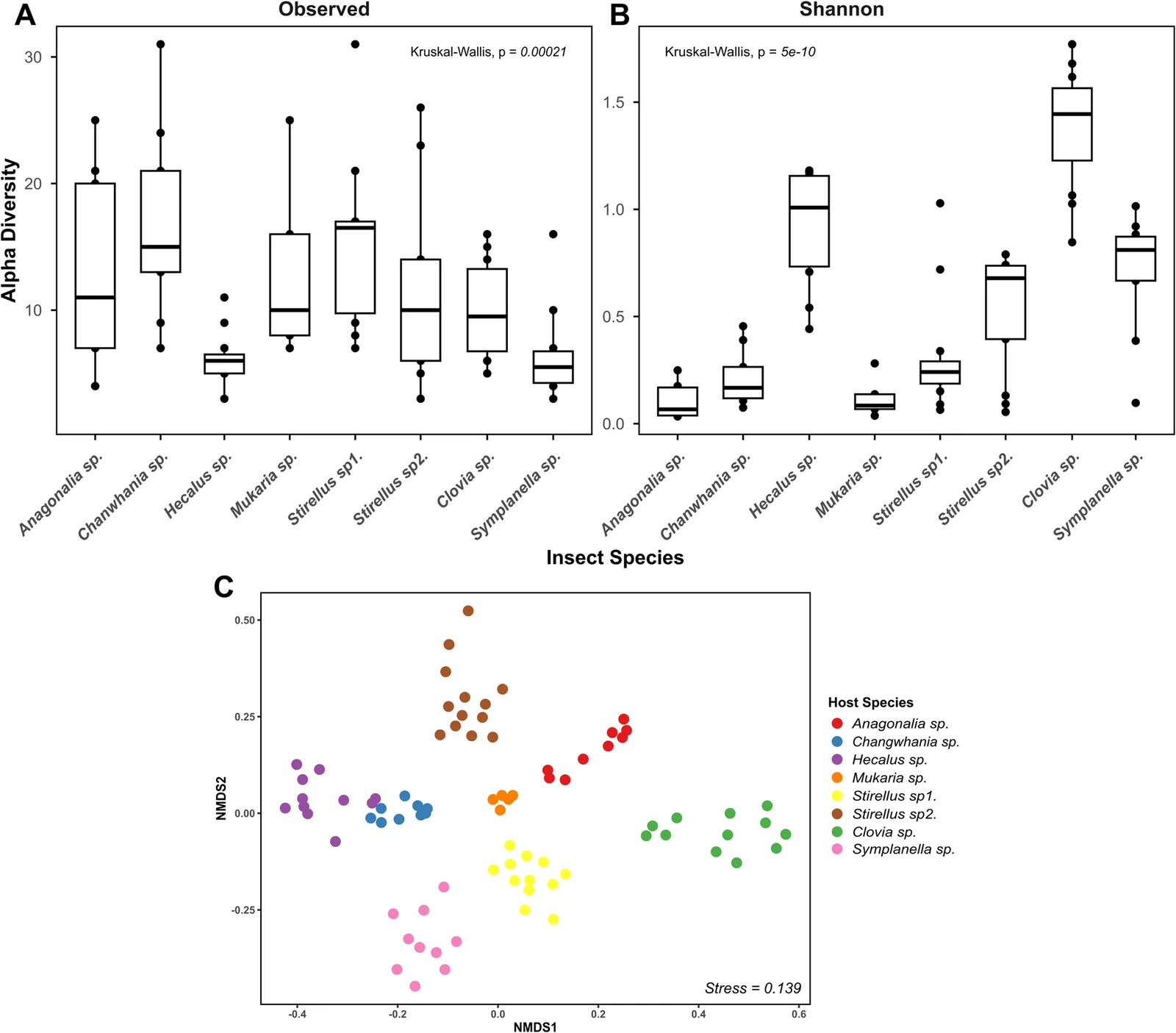
Alpha and Beta Diversity of bacterial communities associated with eight species of Auchenorrhyncha. (**A**) Boxplots of observed ASV richness and (**B**) Shannon diversity index show alpha diversity across different host species. (**C**) NMDS based on Bray-Curtis distances depict beta diversity patterns among individual samples grouped by host species

The analysis of bacterial diversity was normalized by rarefying the data to a sequencing depth of 1,325 reads per sample using ‘*rarefy_even_depth’* function in *phyloseq*, a threshold was chosen to maximize samples retention while standardizing coverage. Alpha (α) diversity indices, including observed (*Obs*) ASV and Shannon index (*H*), were calculated using the *‘estimate_richness’* function in the *vegan* package (v. 2.6.4) [46]. Statistical comparisons of α-diversity among host species were conducted using to estimate microbial richness and evenness. The *Kruskal-Wallis* test was used to compare significant difference among species and pairwise comparisons between species using *Wilcoxon* rank sum test. Beta (β) diversity was evaluated using non-metric multidimensional scaling (NMDS) based on *Bray-Curtis* dissimilarity matrices, using the *‘ordinate’* function in *phyloseq* (Fig. 5). Differences in community structure among host species were tested using permutational multivariate analysis of variance (PERMANOVA) with the ‘*adonis2’* function in *vegan*. Pairwise comparisons among host species were performed using the ‘*pairwise.adonis2’* function. Homogeneity of multivariate dispersion was assessed using ‘*betadisper’* function in *vegan* followed by the permutation tests.

**Fig. 6:**
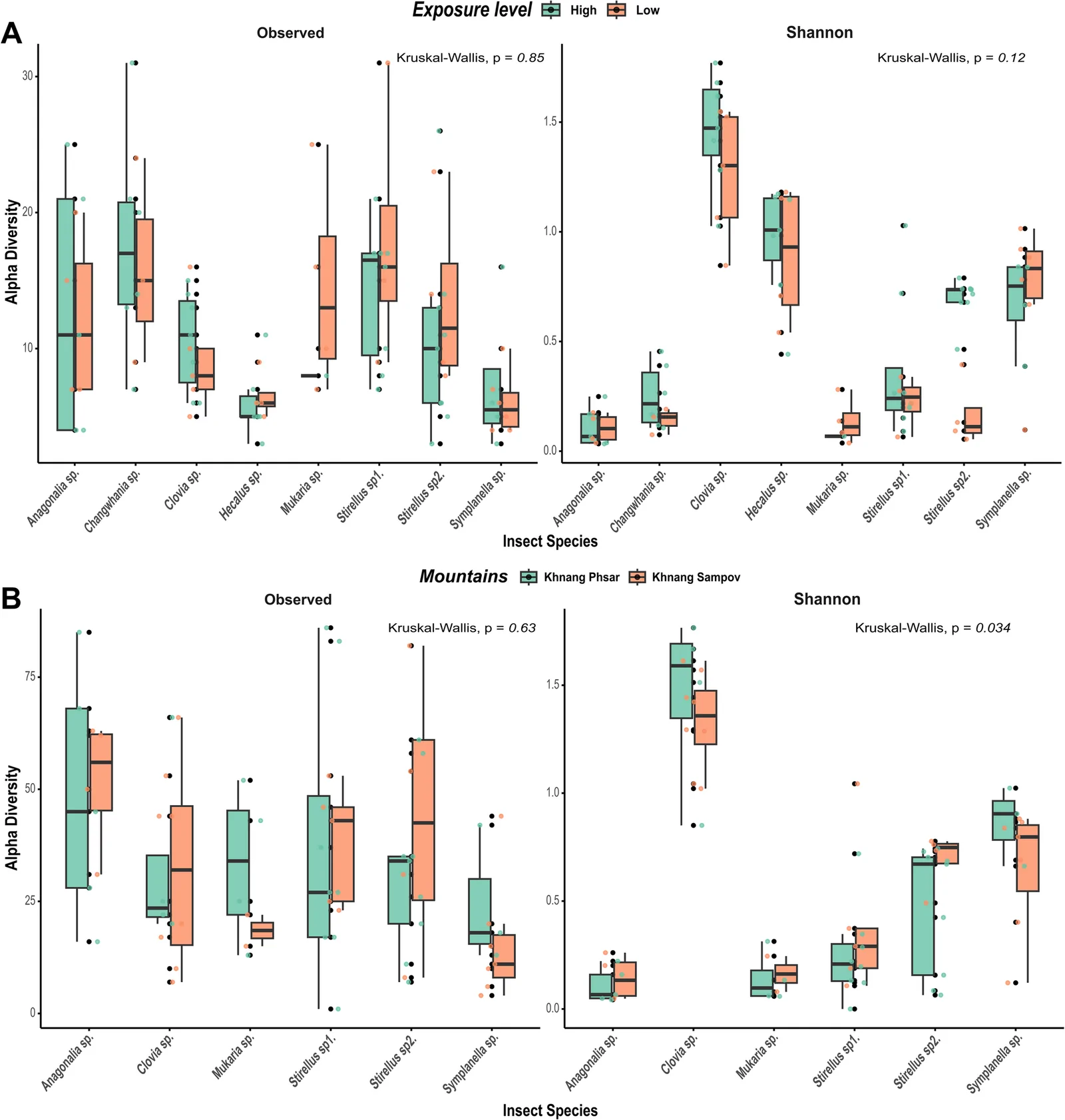
Bacterial communities across Auchenorrhyncha from different upland landscapes of the Central Cardamom Mountains: (**A**) Boxplots showing alpha diversity (observed richness and Shannon index) compared between exposure levels. Colored indicate the two environmental exposures: High exposure (green) and Low exposure (red). (**B**) Boxplots showing alpha diversity compared between the two mountain localities. Colors represent the two sites: Khnang Phsar (green) and Khnang Sampov (red)

To assess the effect of exposure level (low vs. high), influenced by the differences in environmental factors (altitude, sunlight, wind and habitat openness), on the bacterial composition of insect hosts, all auchenorrhynchan species were included in the analysis. In addition, to evaluate variation in bacterial communities between Khnang Phsar and Khnang Sampov mountains, six species (*Anagonalia* sp., *Mukaria* sp., *Stirellus* sp1., *Stirellus* sp2. and *Symplanella* sp.) were selected based on their sufficient and balanced sample sizes, allowing robust comparative analysis. α-diversity indices (*Obs*, *H*) were calculated for both assessments (Fig. 6). Statistical comparisons were performed using the *Kruskal-Wallis*, to evaluate differences between exposure levels and between mountains.

**Fig. 7:**
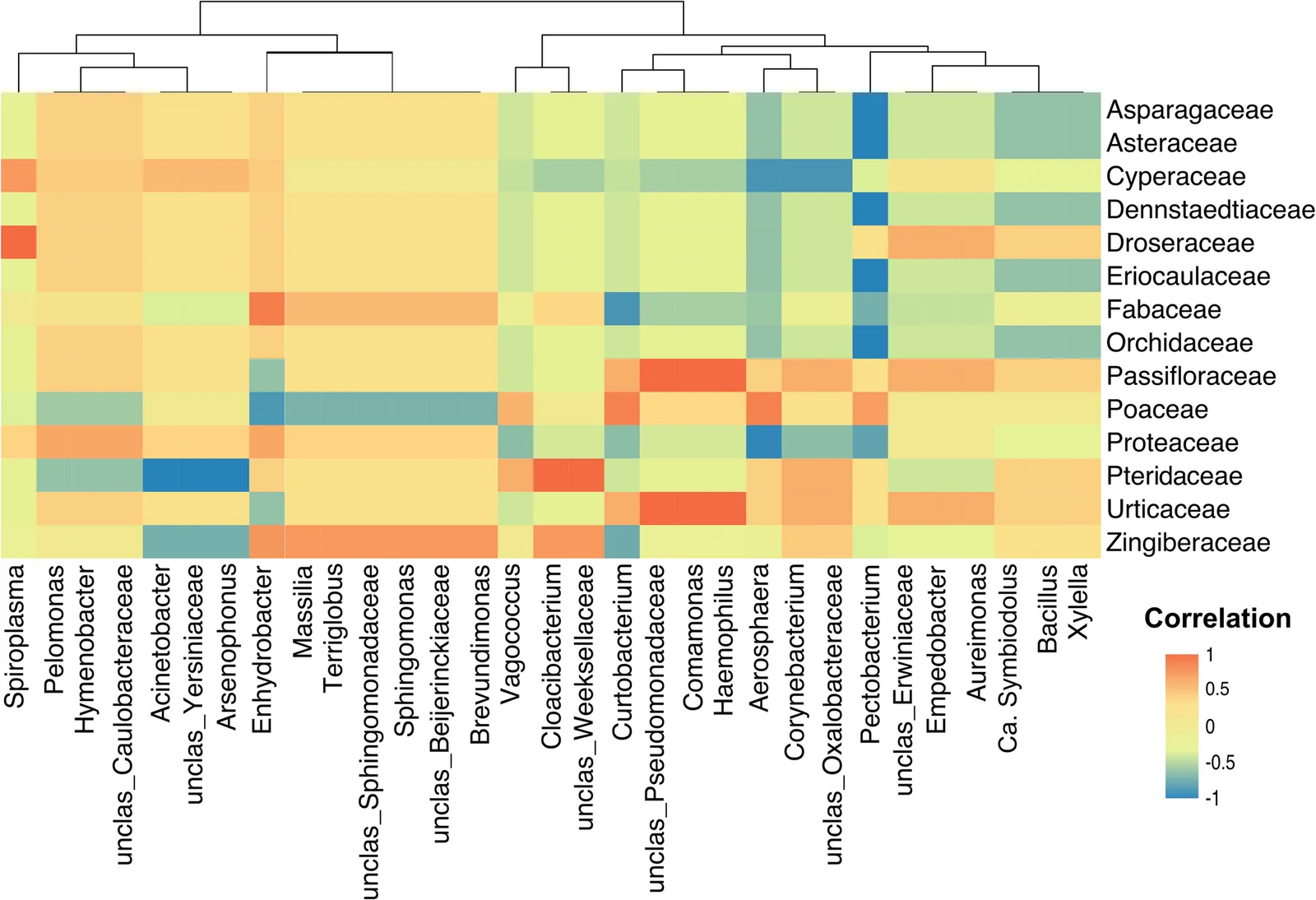
Heatmap of cross-correlations between bacterial genera and associated-plant families across sampling sites. Bacterial genera (x-axis) and plant families (y-axis) were ordered by hierarchical clustering to group taxa with similar correlation profiles. Bacterial abundances were arcsine–square-root transformed prior to analysis, and Spearman’s rank correlations (ρ) were computed between bacterial and plant community matrices. The color scale indicates the strength and direction of correlation (red = strong positive, blue = strong negative, yellow = near zero)

To evaluate the associations between host-plant and bacterial communities, we calculated pairwise correlations between plant family abundances and the relative abundance of bacterial genera across sampling sites. Bacterial genera abundances were arcsine-square-root transformed prior to analysis to account for compositional structure. *Spearman*’s rank coefficients (ρ) were computed using the *‘rcorr’* function in the *Hmisc* R package [47]. Resulting *p-*values were adjusted for multiple testing using the false discovery rate (FDR < 0.05) method for significant associations. Cross-correlation matrices (Fig. 7) for plant and bacterial associations were visualized using the *pheatmap* package [48].

## Results

A total of 1,381,676 raw sequence reads were obtained from the auchenorrhynchan insects after quality control (Table S2). Following sequence filtering, 1,049,624 high-quality reads remained, with an average of 12,646 reads per sample. The assembled paired-end sequences of the 16S rRNA gene had an average length ranging from 402 to 429 bp. All high-quality reads were clustered into 188 ASVs (Table S3).

## Bacterial Composition of Auchenorrhynchan Insects

For 188 bacterial ASVs from the dataset were annotated to 11 phyla, 15 classes, 40 orders, 66 families and 94 genera. Among these, 69.47% of the bacterial composition was classified as Bacteroidota, making it the most dominant phylum, followed by Proteobacteria (29.80%) and Firmicutes (< 1%). At the family level, Blattabacteriaceae (69.45%) was the most dominant, following by Rickettsiaceae (11.03%) and Morganellaceae (7.39%). Several other families each accounted for < 5%, including Anaplasmataceae, Oxalobacteraceae, Fokiniaceae and Pectobacteriaceae. At the genus level, *Karelsulcia* (69.45%) was the most dominant symbiont, followed by *Rickettsia* (11%), with additional symbionts each contributing < 5% such as *Arsenophonus*,* Wolbachia*,* Ca.* Symbiodolus, *Ca.* Zinderia, *Ca.* Lariskella and *Pectobacterium*. Relative abundances of bacterial taxa are shown in Fig. 2 and detailed in supplementary Table S3.

We examined the bacterial composition in specific auchenorrhynchan hosts. *Karelsulcia* was present in all hosts; however, it was highly dominant (> 97%) in Membracoidea species, including *Mukaria* sp., *Anagonalia* sp. and *Changwhania* sp. (Fig. 2 and S3, Table 2). Several host species showed relatively low compositional abundances of *Karelsulcia*, including *Stirellus* sp1. (84.62%), *Stirellus* sp2. (64.97%), *Symplanella* sp. (59.68%), *Clovia* sp. (41.39%) and *Hecalus* sp. (25.99%). In *Clovia* sp., several secondary symbionts were detected, including *Ca.* Lariskella, *Ca.* Zinderia, *Rickettsia*,* Pectobacterium* and *Xylella*. Interestingly, *Rickettsia* (73.82%) was the most abundant in *Hecalus* sp., whereas *Wolbachia* was specifically detected in *Stirellus* sp1. (9.44%), *Symplanella* sp. (12.63%) and *Stirellus* sp2. (13.72%). In addition, *Ca.* Symbiodolus (20.10%) was found in *Stirellus* sp2., while *Arsenophonus* (26.36%) was present in *Symplanella* sp.

**Table 2:** Symbiotic bacteria associated with Auchenorrhyncha hosts of eight species in this study and the number of total reads on average (per sample) and the relative abundance (%) of nine major symbionts in the host species

Superfamily	Host species	*n*	Symbionts										
			Karelsulcia	Arsenophonus	Rickettsia	Spiroplasma	Wolbachia	CandidatusLariskella	CandidatusZinderia	CandidatusSymbiodolus	Pecto-bacterium	Xylella	Others
			9223.33(41.39%)		2839.33(12.70%)			2677.75(12.02%)	5028.42(22.57%)		1531.50(6.87%)	331.75(1.49%)	660(2.96%)
			5336.70(59.68%)	2357.20(26.36%)			1129.30(12.63%)						119.6(1.34%)
Membracoidea	*Anagonalia* sp.	9	16834.33(98.83%)										199.66(1.17%)
	*Changwhania* sp.	9	5360.33(97.82%)										109.33(2.18%)
	*Hecalus* sp.	11	3165.54(25.99%)	3.54(0.03%)	8990.54(73.83%)								19.45(0.16%)
	*Mukaria* sp.	6	6072.16(99.04%)										59.16(0.96%)
	*Stirellus* sp1.	13	9200.46(84.62%)			456(4.19%)	1026.62(9.44%)					1.38(0.13%)	187.77(1.62%)
	*Stirellus* sp2.	13	8900.07(64.97%)				1879.84(13.72%)			2754(20.10%)			164.46(1.20%)

n – number of samples per species

### Symbionts Associated with Auchenorrhyncha

The analysis of primary and secondary symbionts based on the amplicon sequence variants (ASVs) was conducted on eight species of Auchenorrhyncha from the upland landscape of the Central Cardamom Mountains (Fig. 3). While the obligate endosymbiont *Karelsulcia* was dominant across all hosts, the overall composition varied significantly among species. In *Anagonalia* sp., *Changwhania* sp. and *Mukaria* sp. only obligate *Karelsulcia* was detected. Interestingly, no co-obligate symbiont (*Nasuia*) was found in any of the sampled species. In contrast, the co-occurrence of secondary symbionts differed markedly among species (Fig. 4, Table S4). Two secondary symbionts were consistently found in association with *Karelsulcia* across several species. In Membracoidea species, *Arsenophonus* (< 1%) and *Rickettsia* were paired in *Hecalus* sp., while *Wolbachia* co-occurred with *Spiroplasma* and *Ca.* Symbiodolus in *Stirellus* sp1. and *Stirellus* sp2., respectively. In *Symplanella* sp. (Fulgoroidea), both *Arsenophonus* and *Wolbachia* were detected. In contrast, *Clovia* sp. (Cercopoidea) harbored the most diverse assemblage of secondary symbionts, including *Ca.* Lariskella, *Ca.* Zinderia, *Pectobacterium*,* Rickettsia* and *Xylella*.

### Symbiotic Diversity Across Auchenorrhyncha Species

Alpha diversity of auchenorrhynchan species was assessed using observed richness (*Obs*) and the *Shannon-Wiener* diversity index (*H*). We observed higher bacterial richness in *Changwhania* sp. (Membracoidea) based on *Obs*, while *Clovia* sp. (Cercopoidea) showed greater diversity according to the *H* index (Fig. 5A and B). *Kruskal-Wallis* ranks test revealed significant differences in bacterial diversity among insects (*p-value <* 0.05) for both *Obs* and *H* index. In addition, pairwise comparisons using the *Wilcoxon* ranks sum test showed some significant differences between certain species pairs (Table S6). Interestingly, pairwise comparisons of bacterial composition between *Clovia* sp. and other auchenorrhynchan species showed a statistically significant more diverse microbiota in *Clovia* sp.

Beta diversity of bacterial communities was analyzed to determine whether the microbial composition and structure differed across the eight auchenorrhynchan species. Diversity matrix based on the compositional dissimilarity (*Bray-Curtis*) distance, was visualized on the NMDS ordination (Fig. 5C). The NMDS ordination revealed highly distinct clustering among species. In addition, PERMANOVA analysis confirmed significant differences in bacterial communities among species for *Bray-Curtis* distance (R^2^ = 0.87, F = 77.10 *p* = 0.001) (Table 3A). Additionally, pairwise PERMANOVA comparisons revealed significant differences between auchenorrhynchan species (*p* < 0.05).

### The Effect of Topographic Variations on Auchenorrhyncha Bacterial Community

The *Kruskal-Wallis* test of Alpha diversity (*Obs* richness and *H* index) detected no significant differences between exposure levels (Fig. 6A). Pairwise *Wilcoxon* comparison test stratified by species likewise showed no differences, expect for *Stirellus* sp2., which exhibited a higher *H* diversity at low exposure sites (*p* = 0.0028). This pattern is consistent with the detection of the secondary symbiont *Ca.* Symbiodolus in *Stirellus* sp2. at high-exposure sites (Figure S4). In addition, community compositional PERMANOVA results (Table 3A) indicated that exposure level had no significant main effect on bacterial community composition across auchenorrhynchan insects. However, there was a significant interaction dependently between insect hosts and exposure level (R^2^ = 0.015, F = 1.36, *p* = 0.045). Its contribution to overall variation was minor and suggests that the influence of exposure level on microbial communities differs dependently across auchenorrhynchan species.

The bacterial community showed a significant difference in *Shannon* (H) diversity (*p =* 0.034) between the upland landscapes of the Khnang Phsar and Khnang Sampov mountains, whereas *Obs* richness did not differ (Fig. 5B). Pairwise *Wilcoxon* comparison tests confirmed that no species-specific differences were detected between the two landscapes. Moreover, PERMANOVA based on *Bray-Curtis* dissimilarities revealed that community composition did not differ significantly between mountains (R^2^ = 0.002, F = 0.57, *p* = 0.772) or was the evidence of a species-specific dependence and mountains interaction (R^2^ = 0.006, F = 0.43, *p* = 0.997) (Table 3B). These results indicated that, although diversity indices show a modest difference in *H* diversity, there is no evidence that bacterial community composition differs between mountains, either overall or species-dependent manner.

**Table 3:** Summary of PERMANOVA models of Bray-Curtis distances: (**A**) The effects of exposure level on microbial communities across auchenorrhynchan insects and (**B**) Variation in microbial community structure between Khnang Phsar and Khnang Sampov mountains

Variables	Sums of squares	*R* ^2^	F. model	*p*. value
(A) Exposure level of auchenorrhynchan				
InsectSpecies	30.826	0.876	77.100	***0.001***
ExposureLevel	0.097	0.003	1.692	0.098
InsectSpecies: ExposureLevel	0.542	0.015	1.355	***0.045***
Residual	3.713	0.106		
Total	35.177	1.000		
***(B) Mountain’s effect***				
InsectSpecies	22.004	0.855	60.870	***0.001***
Mountains	0.041	0.002	0.572	0.772
InsectSpecies: Mountains	0.158	0.006	0.436	0.997
Residual	3.543	0.137		
Total	25.746	1.000		

Significant *p*-values (< 0.05) are shown in bold and italic

### Significant Associations Between Bacterial and Plant Community

Plant community composition at each sampling site was defined based on the relative abundance and diversity of plant families recorded within sited quadrats. These data were used to represent the dominant plant community structure associated with insect sampling plots. Of the 94 bacterial genera identified, 31 taxa were correlated to 14 plant families.

The cross-correlation analysis revealed distinct patterns of association between bacterial genera and plant community across the upland landscapes (Fig. 7). Both positive and negative correlations were detected, with several clusters of bacteria showing response profiles to particular plant families (Table S7). To ensure that the detected associations between bacterial and plant communities were statistically robust and not driven by random correlation, we applied false discovery rate (FDR) correction (FDR < 0.05) to control for multiple testing. After FDR adjustment, only a restricted subset of bacterial taxa and plant families remained significant. In particular, several facultative symbionts such as *Spiroplasma*,* Cloacibacterium* and *unclassified Weeksellaceae* exhibited strong positive correlation with *Droseraceae* together with *Passifloraceae* and *Urticaceae*, respectively. In contrast, *Pectobacterium* alone displayed negative correlations with *Asparagaceae*,* Asteraceae*,* Dennstaedtiaceae*,* Eriocaulaceae* and *Orchidaceae*. A negative correlation was also observed between the symbiont *Arsenophonus* and the plant family *Pteridaceae.* Additional associations included *Comamonas* and *Haemophilus* with *Asparagaceae*, and *unclassified Pseudomonadaceae* with *Dennstaedtiaceae*. The hierarchical clustering further grouped plant families with similar bacterial association profiles, such as *Eriocaulaceae* and *Orchidaceae*, which clustered together based on shared positive correlations with multiple facultative symbionts.

## Discussion

### Symbiotic Profiles of Auchenorrhyncha

Our study revealed that Auchenorrhyncha from the upland landscapes of the Central Cardamom Mountains harbor a diverse microbiota, with the obligate symbiont *Candidatus* Karelsulcia muelleri dominating all host species (Fig. 2; Table 2). This finding is consistent with previous reports describing *Karelsulcia* as the core nutritional symbiont of Auchenorrhyncha and related lineages [4, 10]. However, while *Karelsulcia* accounted for > 97% of bacterial sequences in some Cicadellidae species (*Anagonalia* sp., *Changwhania* sp. and *Mukaria* sp.), others exhibited substantial contributions from secondary symbionts. Notably, *Hecalus* sp. was dominated by *Rickettsia*, and *Clovia* sp. harbored a particularly diverse assemblage, including *Ca.* Zinderia, *Ca.* Lariskella, *Pectobacterium* and *Xylella.* These differences highlight striking host-specificity in symbiont communities, suggesting variable ecological and evolutionary pressure shaping microbial associations across lineages. Interestingly, no co-obligate *Nasuia* was detected, in contrast to other leafhoppers such as *Nephotettix* or *Macrosteles* of Deltocephalinae, where *Karelsulcia-Nasuia* partnerships are common [20, 22, 49]. The absence of *Nasuia* may indicate lineage-specific symbiont loss or replacement, as reported in other cicadomorph insects [19].

### Distribution of Secondary Symbionts

The results reveal strong interspecific variation in the composition of secondary symbionts among the eight auchenorrhynchan species sampled. The patterns of symbiont occurrence were highly species-specific (Fig. 3). *Arsenophonus* was detected at lower elevation in *Hecalus* sp. compared to the planthopper *Symplanella*. *Arsenophonus* was previously reported to associate with various insects including parasitoid wasps, aphids and leafhoppers [28, 50, 51]. Interestingly, in the two host species (*Hecalus* sp. and *Symplanella* sp.), *Arsenophonus* was associated with different secondary symbionts—*Rickettsia* in *Hecalus* and *Wolbachia* in *Symplanella*. Moreover, *Rickettsia* was found to be more abundant in our study (> 70%) compared to the obligate symbiont *Karelsulcia* (25%) and was detected across all host samples (Table 2). In Japan, *Rickettsia* was reported to infect 31% of the leafhopper *Marosteles striifrons* [16]. The intracellular symbiont *Wolbachia*, which is widespread across arthropod species [52, 53, 54], was also detected in both *Stirellus* host species, although represented by different ASV variants (Fig. 4). *Wolbachia* has likewise been reported in several leafhopper genera, including *Balclutha* [49] and *Exitianus* and *Goniagnathus* [28, 29]. *Arsenophonus*,* Rickettsia*, and *Wolbachia* may contribute to the protection of auchenorrhynchan hosts against pathogens, insecticides, and plants defenses, or influence their capacity to transmit plant pathogens [17, 55].

Interestingly, co-occurrence of *Wolbachia* with *Spiroplasma* in *Stirellus* sp1. and complement presence of *Wolbachia* with *Ca.* Symbiodolus in *Stirellus* sp2. (Fig. 3), could suggest the presence of complex symbiotic assemblages that may perform overlapping or complementary functions. Certain *Spiroplasma* species have been detected in phloem-feeding insect vectors (leafhoppers, psyllids, aphids and others), where they may facilitate the transmission of bacteria between plants [56]. Beyond plant-pathogen interactions, *Spiroplasma* is also well known for its diverse role, ranging from reproductive manipulation such as male-killing [57] to providing protection against parasitoids and fungal pathogens [58]. By contrast, the intracellular symbiont *Ca.* Symbiodolus is vertically transmitted and has been reported in several coleopteran species [59]. Although its role remains unclear, the consistent association of *Ca.* Symbiodolus with different insect groups suggests a potentially broader ecological significance. The co-occurrence of these symbionts in closely related *Stirellus* species highlights the diverse symbiotic strategies in Auchenorrhyncha and raise questions about their roles in host adaptation.

In the spittlebug *Clovia* sp., the symbiotic profile comprised the obligate symbiont *Karelsulcia*, together with the co-obligate *Ca.* Zinderia [60] and several secondary symbionts (Fig. 3). The detection of this co-obligate symbiont has been described almost exclusively in spittlebugs (Cercopoidea), where it complements *Karelsulcia* by providing essential nutrients absent from the insect diet [61, 62]. A particularly noteworthy finding is the consistent detection of *Ca.* Lariskella, in all samples of *Clovia* sp., representing, to our knowledge, the first record of this symbiont in Cambodian auchenorrhynchan insects. This Alphaproteobacterium has previously been described in stink bugs, where it is hypothesized to contribute to host nutrients [24, 63]. In addition, the presence of complementary secondary symbionts, such as *Pectobacterium* and *Xylella*, both plant-associated phytopathogens previously linked to *Philaenus* spittlebugs [64, 65], suggests potential ecological connections between insect feeding, plant pathogens, and symbiont communities in Auchenorrhyncha. Together, these findings highlight the dynamic and host-specific nature of secondary symbiont associations in Auchenorrhyncha, pointing to their potential roles in insect-plant interactions and symbiont-mediated adaptations.

### Symbiotic Communities and their Influence Factors

Our results revealed clear interspecific variation in symbiotic diversity across the eight Auchenorrhyncha species examined (Fig. 5). Alpha diversity patterns differed depending on the diversity matric: *Changwhania* sp. exhibited the highest bacterial richness (Observed ASVs), while *Clovia* sp. harbored the most diverse community based on *Shannon* (H) diversity, reflecting greater evenness in its bacterial composition. Pairwise comparisons indicated that *Clovia* sp. maintained a significantly more diverse microbiota than several other species (Table S6). This aligns with previous findings showing that different auchenorrhynchan lineages can host distinct symbiont assemblages shaped by their evolutionary history and ecological niches [8, 66]. Beta diversity analysis further supported strong host-specific structuring of bacterial communities. NMDS ordination-based *Bray-Curtis* distances revealed clear clustering of samples by insect species, and PERMANOVA confirmed that host species explained 87% of the variation. These results are consistent with previous studies in leafhoppers where microbiota composition was tightly associated with host phylogeny and diet [19, 67].

In contrast to the strong host-specific patterns, the influence of environmental factors such as exposure level and mountain locality on symbiotic diversity was limited (Fig. 6). Pairwise comparison did not show significant differences between exposure level, except in *Stirellus* sp2., which displayed higher *Shannon* diversity under high exposure conditions—a pattern that coincided with the detection of the secondary symbiont *Ca.* Symbiodolus. This suggests that microhabitat variation may shape bacterial community structure in certain species. A similar pattern was observed in other *Stirellus* species, where variation in *Wolbachia* dominance occurred across host populations [29]. Nevertheless, PERMANOVA indicated that exposure level explained only a minor proportion of the variation (0.03%), with a small but significant host-exposure level association (0.15%) (Table 3A). Likewise, although *Shannon* diversity was marginally higher in samples from upland landscape of Khnang Sampov compared to Khnang Phsar, community composition did not differ significantly between the two mountain sites (Fig. 6B, S4). Both PERMANOVA and pairwise comparisons showed no evidence of species-specific effects across landscapes. This pattern is consistent with other insect-microbe systems, where host phylogeny and physiology outweigh geographic or habitat effects in structuring microbial communities [24, 66, 67, 68]. Similar stability of microbiomes across geographical and ecological gradients have been reported in *Scaphoideus titanus* [27], *Macrosteles* leafhoppers [24], and *Philaenus* spittlebugs [65]. Taken together, these findings demonstrate that the microbial communities of Auchenorrhyncha is primarily governed by host species rather than environmental gradients.

### Plant-symbionts Association in Auchenorrhyncha Insects

The cross-correlation analysis showed that only a restricted subset of bacterial genera exhibited significant associations with plant families, highlighting the selective role of vegetation community composition in structuring Auchenorrhyncha microbiotas (Fig. 7). For example, *Spiroplasma* showed a strong positive association with sundew plants (*Droseraceae*), suggesting that certain plant groups may create ecological conditions that favor the persistence of specific facultative symbionts within *Stirellus* sp1. at the high-exposure sampling site (KPS04). Such associations may arise through shared feeding niches or plant-derived metabolites. Although *Spiroplasma* has been reported from a wide range of arthropod species as well as vegetation [56], there is currently no direct evidence of its presence in *Droseraceae*. Conversely, negative correlations of the symbionts *Arsenophonus* and *Pectobacterium* indicated that some plant lineages may act as ecological filters, restricting or reducing the prevalence of these symbiotic associations [30]. Although most bacterial genera exhibited weak or inconsistent associations, the significant correlations highlight the potential of plant communities to structure insect-associated microbiota. However, since the associations of microbiota are with the plant communities and not observed individual insect-plant interactions, the results should be interpreted cautiously. Overall, these results emphasize that while host species remains the dominant factor shaping bacterial communities in Auchenorrhyncha [65, 68], plant community composition also play a selective role in modulating assemblages, with possible consequences for host ecology, nutrition and vector competence [69, 70].

## Conclusion

The study reveals that symbiont communities of upland Auchenorrhyncha insects at the Central Cardamom mountains are strongly structured by host species, with obligate symbiont *Karelsulcia* present, but accompanied by remarkable variation in secondary symbionts across species. Environmental gradients contributed little to the overall microbiota composition, although localized effects, such as occurrence of *Ca.* Symbiodolus in *Stirellus* sp2., were detected. Plant-microbe associations further suggest selective filtering of secondary symbionts by particular plant lineages. Together, these findings highlight the dominant role of host species in shaping symbiont diversity, while also emphasizing ecological interactions that may influence insect adaptation, nutritional ecology, and pathogen transmission in tropical landscapes. However, the interactive effect of host species, plant associations, and geographic context could not be explicitly resolved using causal or multivariate modeling approaches (e.g., structural equation modelling). In addition, the use of 16S rRNA amplicon sequencing limits functional inference. Future research integrating metagenomic or transcriptomic approaches, combined with experimental feeding assays across different plant lineages and environmental gradients, will be essential to explain how host, plant, and environmental factors jointly structure symbiont communities and to elucidate the functional roles of secondary symbionts [32].

## Supplementary Information

Below is the link to the electronic supplementary material.

Supplementary Material 1

Supplementary Material 2

## Data Availability

The raw bacterial 16S rRNA (V3-V4) gene sequence reads were deposited in the European Nucleotide Archive (ENA accession *PRJEB87188* ).

## References

[1] Cao Y, Dietrich CH (2021) Phylogenomics of flavobacterial insect nutritional endosymbionts with implications for Auchenorrhyncha phylogeny. Cladistics 38(1):38–58. 10.1111/cla.12474 35049085 10.1111/cla.12474

[2] Huang Z, Zhou J, Zhang Z, He H, Wei C (2023) A Study on Symbiotic Systems of Cicadas Provides New Insights into Distribution of Microbial Symbionts and Improves Fluorescence In Situ Hybridization Technique. Int J Mol Sci 24(3):2434. 10.3390/ijms24032434 36768757 10.3390/ijms24032434 PMC9917331

[3] Wangkeeree J, Miller TA, Hanboonsong Y (2012) Candidates for Symbiotic Control of Sugarcane White Leaf Disease. Appl Environ Microbiol 78(19):6804–6811. 10.1128/AEM.01439-12 22798373 10.1128/AEM.01439-12 PMC3457511

[4] Kobiałka M, Świerczewski D, Walczak M, Urbańczyk W (2025) Extremely distinct microbial communities in closely related leafhopper subfamilies: Typhlocybinae and Eurymelinae (Cicadellidae, Hemiptera). mSystems 10(7):e00603–e00625. 10.1128/msystems.00603-25 40569073 10.1128/msystems.00603-25 PMC12282065

[5] Mao M, Bennett GM (2020) Symbiont replacements reset the co-evolutionary relationship between insects and their heritable bacteria. ISME J 14(6):1384–1395. 10.1038/s41396-020-0616-4 32076126 10.1038/s41396-020-0616-4 PMC7242365

[6] Wang D, He H, Wei C (2023) Cellular and potential molecular mechanisms underlying transovarial transmission of the obligate symbiont *Sulcia* in cicadas. Environ Microbiol 25(4):836–852. 10.1111/1462-2920.16310 36515176 10.1111/1462-2920.16310

[7] Michalik A, Jankowska W, Kot M, Gołas A, Szklarzewicz T (2014) Symbiosis in the green leafhopper, *Cicadella viridis* (Hemiptera, Cicadellidae). Association *in statu nascendi*? Arthropod Struct Dev 43(6):579–587. 10.1016/j.asd.2014.07.005 25102427 10.1016/j.asd.2014.07.005

[8] Michalik A, Castillo Franco D, Kobiałka M, Szklarzewicz T, Stroiński A, Łukasik P (2021) Alternative Transmission Patterns in Independently Acquired Nutritional Cosymbionts of Dictyopharidae Planthoppers. mBio 12(4):e01228–e01221. 10.1128/mBio.01228-21 34465022 10.1128/mBio.01228-21 PMC8406288

[9] Moriyama M, Fukatsu T (2022) Host’s demand for essential amino acids is compensated by an extracellular bacterial symbiont in a hemipteran insect model. Front Physiol 13:1028409. 10.3389/fphys.2022.1028409 36246139 10.3389/fphys.2022.1028409 PMC9561257

[10] Moran NA, Tran P, Gerardo NM (2005) Symbiosis and Insect Diversification: An Ancient Symbiont of Sap-Feeding Insects from the Bacterial Phylum *Bacteroidetes*. Appl Environ Microbiol 71(12):8802–8810. 10.1128/AEM.71.12.8802-8810.2005 16332876 10.1128/AEM.71.12.8802-8810.2005 PMC1317441

[11] Ge Y, Jing Z, Diao Q, He J-Z, Liu Y-J (2021) Host Species and Geography Differentiate Honeybee Gut Bacterial Communities by Changing the Relative Contribution of Community Assembly Processes. mBio 12(3):e00751–e00721. 10.1128/mBio.00751-21 34061602 10.1128/mBio.00751-21 PMC8262996

[12] Zhou J, Ning D (2017) Stochastic Community Assembly: Does It Matter in Microbial Ecology? Microbiol Mol Biol Rev 81(4):e00002–17. 10.1128/MMBR.00002-17 29021219 10.1128/MMBR.00002-17 PMC5706748

[13] Zhu Y, Yang R, Wang X, Wen T, Gong M, Shen Y, Xu J, Zhao D, Du Y (2022) Gut microbiota composition in the sympatric and diet-sharing *Drosophila simulans* and *Dicranocephalus wallichii bowringi* shaped largely by community assembly processes rather than regional species pool. iMeta 1(4):e57. 10.1002/imt2.57 38867909 10.1002/imt2.57 PMC10989964

[14] Bennett GM, Abbà S, Kube M, Marzachì C (2016) Complete Genome Sequences of the Obligate Symbionts *Candidatus* Sulcia muelleri and *Ca.* Nasuia deltocephalinicola from the Pestiferous Leafhopper *Macrosteles quadripunctulatus* (Hemiptera: Cicadellidae). Genome Announcements 4(1):e01604–e01615. 10.1128/genomeA.01604-15 26798106 10.1128/genomeA.01604-15 PMC4722273

[15] Oren A, Garrity GM, Parker CT, Chuvochina M, Trujillo ME (2020) Lists of names of prokaryotic Candidatus taxa. Int J Syst Evol MicroBiol 70(7):3956–4042. 10.1099/ijsem.0.003789 32603289 10.1099/ijsem.0.003789

[16] Ishii Y, Matsuura Y, Kakizawa S, Nikoh N, Fukatsu T (2013) Diversity of Bacterial Endosymbionts Associated with Macrosteles Leafhoppers Vectoring Phytopathogenic Phytoplasmas. Appl Environ Microbiol 79(16):5013–5022. 10.1128/AEM.01527-13 23770905 10.1128/AEM.01527-13 PMC3754707

[17] Cooper WR, Walker WB, Angelella GM, Grimm S, Foutz KD, Harper JJ, Nottingham SJ, Northfield LB, Wohleb TD, C. H., Strausbaugh CA (2023) Bacterial Endosymbionts Identified From Leafhopper (Hemiptera: Cicadellidae) Vectors of Phytoplasmas. Environ Entomol 52(2):243–253. 10.1093/ee/nvad015 36869841 10.1093/ee/nvad015

[18] Wu W, Lei J-N, Mao Q, Tian Y-Z, Shan H-W, Chen J-P (2023) Distribution, Vertical Transmission, and Cooperative Mechanisms of Obligate Symbiotic Bacteria in the Leafhopper *Maiestas dorsalis* (Hemiptera, Cicadellidea). Insects 14(8):710. 10.3390/insects14080710 37623420 10.3390/insects14080710 PMC10455556

[19] Mao M, Yang X, Poff K, Bennett G (2017) Comparative Genomics of the Dual-Obligate Symbionts from the Treehopper, *Entylia carinata* (Hemiptera: Membracidae), Provide Insight into the Origins and Evolution of an Ancient Symbiosis. Genome Biol Evol 9(6):1803–1815. 10.1093/gbe/evx134 28854637 10.1093/gbe/evx134 PMC5533117

[20] Moriyama M, Nishide Y, Toyoda A, Itoh T, Fukatsu T (2023) Complete genomes of mutualistic bacterial co-symbionts *Candidatus* Sulcia muelleri and *Candidatus* Nasuia deltocephalinicola of the rice green leafhopper *Nephotettix cincticeps*. Microbiol Resource Announcements 12(9):e00353–e00323. 10.1128/MRA.00353-23 10.1128/MRA.00353-23 PMC10508130 37623315

[21] Kobiałka M, Michalik A, Walczak M, Szklarzewicz T (2018) Dual Bacterial-Fungal Symbiosis in Deltocephalinae Leafhoppers (Insecta, Hemiptera, Cicadomorpha: Cicadellidae). Microb Ecol 75(3):771–782. 10.1007/s00248-017-1075-y 28939987 10.1007/s00248-017-1075-y PMC5856902

[22] Kobiałka M, Michalik A, Walczak M, Junkiert Ł, Szklarzewicz T (2016) Sulcia symbiont of the leafhopper Macrosteles laevis (Ribaut, 1927) (Insecta, Hemiptera, Cicadellidae: Deltocephalinae) harbors Arsenophonus bacteria. Protoplasma 253(3):903–912. 10.1007/s00709-015-0854-x 26188921 10.1007/s00709-015-0854-x PMC4819937

[23] Waneka G, Vasquez YM, Bennett GM, Sloan DB (2020) Mutational pressure drives differential genome conservation in two bacterial endosymbionts of sap feeding insects [Preprint]. Genetics. 10.1101/2020.07.29.225037 10.1093/gbe/evaa254 PMC7952229 33275136

[24] Mulio SÅ, Zwolińska A, Klejdysz T, Prus-Frankowska M, Michalik A, Kolasa M, Łukasik P (2024) Limited variation in microbial communities across populations of *Macrosteles* leafhoppers (Hemiptera: Cicadellidae). Environ Microbiol Rep 16(3):e13279. 10.1111/1758-2229.13279 38855918 10.1111/1758-2229.13279 PMC11163331

[25] Trevelline BK, Fontaine SS, Hartup BK, Kohl KD (2019) Conservation biology needs a microbial renaissance: A call for the consideration of host-associated microbiota in wildlife management practices. *Proceedings of the Royal Society B: Biological Sciences*, *286*(1895), 20182448. 10.1098/rspb.2018.2448 10.1098/rspb.2018.2448 PMC6364583 30963956

[26] Crotti E, Rizzi A, Chouaia B, Ricci I, Favia G, Alma A, Sacchi L, Bourtzis K, Mandrioli M, Cherif A, Bandi C, Daffonchio D (2010) Acetic Acid Bacteria, Newly Emerging Symbionts of Insects. Appl Environ Microbiol 76(21):6963–6970. 10.1128/AEM.01336-10 20851977 10.1128/AEM.01336-10 PMC2976266

[27] Enciso JS, Corretto E, Borruso L, Schuler H (2024) Limited Variation in Bacterial Communities of *Scaphoideus titanus* (Hemiptera: Cicadellidae) Across European Populations and Different Life Stages. Insects 15(11):830. 10.3390/insects15110830 39590429 10.3390/insects15110830 PMC11595099

[28] Phauk S, Assentato L, Meas S, Terenius O (2025) Primary and Secondary Symbionts of Cambodian Cicadellidae and the Role of Parasitisation. Environ Microbiol Rep 17(5):e70196. 10.1111/1758-2229.70196 40957832 10.1111/1758-2229.70196 PMC12440678

[29] Phauk S, Assentato L, Sin S, Uk O, Hap S, Terenius O (2025) Symbiont Diversity of Rice-Associated Leafhoppers (Cicadellidae) in the Tropical Floodplains of the Tonle Sap Lake, Cambodia. Microb Ecol 88(1):109. 10.1007/s00248-025-02619-9 41105260 10.1007/s00248-025-02619-9 PMC12534257

[30] Douglas AE (2015) Multiorganismal Insects: Diversity and Function of Resident Microorganisms. Ann Rev Entomol 60(1):17–34. 10.1146/annurev-ento-010814-020822 25341109 10.1146/annurev-ento-010814-020822 PMC4465791

[31] Sin S, Khin C, Chhorn S, Yok G, Phak S, Thou S, Phauk S (2021) First record of the carrion beetle *Diamesus osculans* (Vigors, 1825) (Coleoptera: Silphidae) in Cambodia. Cambodian J Nat History 2021(1):8–11

[32] Buck M, Nilsson LKJ, Brunius C, Dabiré RK, Hopkins R, Terenius O (2016) Bacterial associations reveal spatial population dynamics in *Anopheles gambiae* mosquitoes. Sci Rep 6(1):22806. 10.1038/srep22806 26960555 10.1038/srep22806 PMC4785398

[33] Herlemann DP, Labrenz M, Jürgens K, Bertilsson S, Waniek JJ, Andersson AF (2011) Transitions in bacterial communities along the 2000 km salinity gradient of the Baltic Sea. ISME J 5(10):1571–1579. 10.1038/ismej.2011.41 21472016 10.1038/ismej.2011.41 PMC3176514

[34] Sinclair L, Osman OA, Bertilsson S, Eiler A (2015) Microbial Community Composition and Diversity via 16S rRNA Gene Amplicons: Evaluating the Illumina Platform. PLoS ONE 10(2):e0116955. 10.1371/journal.pone.0116955 25647581 10.1371/journal.pone.0116955 PMC4315398

[35] Martin M (2011) Cutadapt removes adapter sequences from high-throughput sequencing reads. EMBnet J 17(1):10. 10.14806/ej.17.1.200

[36] Krueger F, James F, Ewels P, Afyounian E, Weinstein M, Schuster-Boeckler B, Hulselmans G, Sclamons (2023) *FelixKrueger/TrimGalore: V0.6.10 - add default decompression path* (Version 0.6.10) [Computer software]. [object Object]. 10.5281/ZENODO.5127898

[37] Callahan BJ, McMurdie PJ, Rosen MJ, Han AW, Johnson AJA, Holmes SP (2016) DADA2: High-resolution sample inference from Illumina amplicon data. Nat Methods 13(7):581–583. 10.1038/nmeth.3869 27214047 10.1038/nmeth.3869 PMC4927377

[38] Wright ES (2016) Using DECIPHER v2.0 to Analyze Big Biological Sequence Data in R. R J 8(1):352–359

[39] Murali A, Bhargava A, Wright ES (2018) IDTAXA: A novel approach for accurate taxonomic classification of microbiome sequences. Microbiome 6(1):140. 10.1186/s40168-018-0521-5 30092815 10.1186/s40168-018-0521-5 PMC6085705

[40] Katoh K, Standley DM (2013) MAFFT Multiple Sequence Alignment Software Version 7: Improvements in Performance and Usability. Mol Biol Evol 30(4):772–780. 10.1093/molbev/mst010 23329690 10.1093/molbev/mst010 PMC3603318

[41] Nguyen L-T, Schmidt HA, Von Haeseler A, Minh BQ (2015) IQ-TREE: A Fast and Effective Stochastic Algorithm for Estimating Maximum-Likelihood Phylogenies. Mol Biol Evol 32(1):268–274. 10.1093/molbev/msu300 25371430 10.1093/molbev/msu300 PMC4271533

[42] McMurdie PJ, Holmes S (2013) phyloseq: An R Package for Reproducible Interactive Analysis and Graphics of Microbiome Census Data. PLoS ONE 8(4):e61217. 10.1371/journal.pone.0061217 23630581 10.1371/journal.pone.0061217 PMC3632530

[43] Wickham H (2016) ggplot2: Elegant Graphics for Data Analysis. Springer-, New York. https://ggplot2.tidyverse.org

[44] Gu Z (2022) Complex heatmap visualization. iMeta 1(3):e43. 10.1002/imt2.43 38868715 10.1002/imt2.43 PMC10989952

[45] Letunic I, Bork P (2024) Interactive Tree of Life (iTOL) v6: Recent updates to the phylogenetic tree display and annotation tool. Nucleic Acids Res 52(W1):W78–W82. 10.1093/nar/gkae268 38613393 10.1093/nar/gkae268 PMC11223838

[46] Oksanen J, Simpson GL, Blanchet FG, Kindt R, Legendre P, Minchin PR, O’Hara RB, Solymos P, Stevens MHH, Szoecs E, Wagner H, Barbour M, Bedward M, Bolker B, Daniel B. Carvalho G, Chirico M, De Caceres M, Durand S, … Weedon J (2022). vegan: Community Ecology Package. Vegan: Community Ecology Package. https://cran.r-project.org/web/packages/vegan/index.html

[47] Harrell Jr, Frank E (2024) *Hmisc: Harrell Miscellaneous*. https://CRAN.R-project.org/package=Hmisc

[48] Kolde R (2019) *pheatmap: Pretty Heatmaps*. R package version 1.0.12. Available at: https://CRAN.R-project.org/package=pheatmap

[49] Kobiałka M, Michalik A, Szwedo J, Szklarzewicz T (2018) Diversity of symbiotic microbiota in Deltocephalinae leafhoppers (Insecta, Hemiptera, Cicadellidae). Arthropod Struct Dev 47(3):268–278. 10.1016/j.asd.2018.03.005 29621609 10.1016/j.asd.2018.03.005

[50] Bressan A (2014) Emergence and evolution of Arsenophonus bacteria as insect-vectored plant pathogens. Infect Genet Evol 22:81–90. 10.1016/j.meegid.2014.01.004 24444593 10.1016/j.meegid.2014.01.004

[51] Nováková E, Hypša V, Moran NA (2009) Arsenophonus, an emerging clade of intracellular symbionts with a broad host distribution. BMC Microbiol 9(1):143. 10.1186/1471-2180-9-143 19619300 10.1186/1471-2180-9-143 PMC2724383

[52] Guo H, Wang N, Niu H, Zhao D, Zhang Z (2021) Interaction of *Arsenophonus* with *Wolbachia* in *Nilaparvata lugens*. BMC Ecol Evol 21(1):31. 10.1186/s12862-021-01766-0 33610188 10.1186/s12862-021-01766-0 PMC7896400

[53] Sazama EJ, Bosch MJ, Shouldis CS, Ouellette SP, Wesner JS (2017) Incidence of *Wolbachia* in aquatic insects. Ecol Evol 7(4):1165–1169. 10.1002/ece3.2742 28303186 10.1002/ece3.2742 PMC5306009

[54] Zhu Y-X, Song Y-L, Zhang Y-K, Hoffmann AA, Zhou J-C, Sun J-T, Hong X-Y (2018) Incidence of Facultative Bacterial Endosymbionts in Spider Mites Associated with Local Environments and Host Plants. Appl Environ Microbiol 84(6):e02546–e02517. 10.1128/AEM.02546-17 29330177 10.1128/AEM.02546-17 PMC5835729

[55] Liu H, Zhao D, Niu H, Zhang Z, Wang N, Liu X, Guo H (2024) *Arsenophonus* and *Wolbachia*-mediated insecticide protection in *Nilaparvata lugens*. J Pest Sci. 10.1007/s10340-024-01810-0

[56] Cisak E, Wójcik-Fatla A, Zając V, Sawczyn A, Sroka J, Dutkiewicz J (2015) *Spiroplasma* – an emerging arthropod-borne pathogen? Ann Agric Environ Med 22(4):589–593. 10.5604/12321966.1185758 26706960 10.5604/12321966.1185758

[57] Haselkorn TS, Markow TA, Moran NA (2009) Multiple introductions of the *Spiroplasma* bacterial endosymbiont into *Drosophila*. Mol Ecol 18(6):1294–1305. 10.1111/j.1365-294X.2009.04085.x 19226322 10.1111/j.1365-294X.2009.04085.x

[58] Xie J, Butler S, Sanchez G, Mateos M (2014) Male killing *Spiroplasma* protects *Drosophila melanogaster* against two parasitoid wasps. Heredity 112(4):399–408. 10.1038/hdy.2013.118 24281548 10.1038/hdy.2013.118 PMC3966124

[59] Wierz JC, Dirksen P, Kirsch R, Krüsemer R, Weiss B, Pauchet Y, Engl T, Kaltenpoth M (2024) Intracellular symbiont *Symbiodolus* is vertically transmitted and widespread across insect orders. ISME J 18(1):wrae099. 10.1093/ismejo/wrae099 38874172 10.1093/ismejo/wrae099 PMC11322605

[60] Koga R, Bennett GM, Cryan JR, Moran NA (2013) Evolutionary replacement of obligate symbionts in an ancient and diverse insect lineage. Environ Microbiol 15(7):2073–2081. 10.1111/1462-2920.12121 23574391 10.1111/1462-2920.12121

[61] Bennett GM, Moran NA (2013) Small, Smaller, Smallest: The Origins and Evolution of Ancient Dual Symbioses in a Phloem-Feeding Insect. Genome Biol Evol 5(9):1675–1688. 10.1093/gbe/evt118 23918810 10.1093/gbe/evt118 PMC3787670

[62] McCutcheon JP, Moran NA (2012) Extreme genome reduction in symbiotic bacteria. Nat Rev Microbiol 10(1):13–26. 10.1038/nrmicro2670 10.1038/nrmicro2670 22064560

[63] Matsuura Y, Kikuchi Y, Meng XY, Koga R, Fukatsu T (2012) Novel Clade of Alphaproteobacterial Endosymbionts Associated with Stinkbugs and Other Arthropods. Appl Environ Microbiol 78(12):4149–4156. 10.1128/AEM.00673-12 22504806 10.1128/AEM.00673-12 PMC3370525

[64] Cornara D, Saponari M, Zeilinger AR, De Stradis A, Boscia D, Loconsole G, Bosco D, Martelli GP, Almeida RPP, Porcelli F (2017) Spittlebugs as vectors of *Xylella fastidiosa* in olive orchards in Italy. J Pest Sci 90(2):521–530. 10.1007/s10340-016-0793-0 10.1007/s10340-016-0793-0 PMC5320020 28275326

[65] Kolasa M, Kajtoch Ł, Michalik A, Maryańska-Nadachowska A, Łukasik P (2023) Till evolution do us part: The diversity of symbiotic associations across populations of *Philaenus* spittlebugs. Environ Microbiol 25(11):2431–2446. 10.1111/1462-2920.16473 37525959 10.1111/1462-2920.16473

[66] Michalik A, Franco DC, Deng J, Szklarzewicz T, Stroiński A, Kobiałka M, Łukasik P (2023) Variable organization of symbiont-containing tissue across planthoppers hosting different heritable endosymbionts. Front Physiol 14:1135346. 10.3389/fphys.2023.1135346 37035661 10.3389/fphys.2023.1135346 PMC10073718

[67] Sudakaran S, Salem H, Kost C, Kaltenpoth M (2012) Geographical and ecological stability of the symbiotic mid-gut microbiota in European firebugs, *P yrrhocoris apterus* (Hemiptera, P yrrhocoridae). Mol Ecol 21(24):6134–6151. 10.1111/mec.12027 23017151 10.1111/mec.12027

[68] Jones RT, Sanchez LG, Fierer N (2013) A Cross-Taxon Analysis of Insect-Associated Bacterial Diversity. PLoS ONE 8(4):e61218. 10.1371/journal.pone.0061218 23613815 10.1371/journal.pone.0061218 PMC3628706

[69] Hammer TJ, Moran NA (2019) Links between metamorphosis and symbiosis in holometabolous insects. Philosophical Trans Royal Soc B: Biol Sci 374(1783):20190068. 10.1098/rstb.2019.0068 10.1098/rstb.2019.0068 PMC6711286 31438811

[70] Vacher C, Hampe A, Porté AJ, Sauer U, Compant S, Morris CE (2016) The Phyllosphere: Microbial Jungle at the Plant–Climate Interface. Annu Rev Ecol Evol Syst 47(1):1–24. 10.1146/annurev-ecolsys-121415-032238

