# Supplementary material for: Symbiotic Diversity of Sap-Feeding Auchenorrhyncha (Hemiptera) in the Upland Landscapes of Central Cardamom Mountains, Cambodia
Source: Microb Ecol. 2026 Feb 28;89:71. doi: 10.1007/s00248-026-02724-3 (PMC12999766; doi:10.1007/s00248-026-02724-3)
Supplement: Supplementary file 1 [file 248_2026_2724_MOESM1_ESM.docx]

***Symbiotic diversity of sap-feeding Auchenorrhyncha (Hemiptera) in the upland landscapes of Central Cardamom Mountains, Cambodia***

**Sophany Phauk^1,2^*, Sopha Sin^3^ and Olle Terenius^1^**

^1^	Department of Cell and Molecular Biology, Microbiology, Uppsala University, Uppsala, Sweden

^2^	Department of Biology, Faculty of Science, Royal University of Phnom Penh, Cambodia

^3^	Centre for Biodiversity Conservation, Faculty of Science, Royal University of Phnom Penh, Cambodia

*	Corresponding author

Email address: [sophany.phauk@icm.uu.se](mailto:sophany.phauk@icm.uu.se)

**Table S1:** Metadata and information of the dataset

| **SampleID** | **Superfamily** | **Insect Species** | **Sex** | **Altitude** | **Mountains** | **Peaks** | **Sample Site** | **Collection Date** |
| --- | --- | --- | --- | --- | --- | --- | --- | --- |
| KS01 | Membracoidea | *Stirellus* sp1. | M | Low | Khnang Sampov | Lower peak | KSP01 | 12 August 2020 |
| KS02 | Membracoidea | *Stirellus* sp1. | F | Low | Khnang Sampov | Lower peak | KSP01 | 12 August 2020 |
| KS03 | Membracoidea | *Stirellus* sp1. | F | Low | Khnang Sampov | Lower peak | KSP01 | 12 August 2020 |
| KS04 | Fulgoroidea | *Symplanella* sp. | M | Low | Khnang Sampov | Lower peak | KSP01 | 12 August 2020 |
| KS05 | Fulgoroidea | *Symplanella* sp. | F | Low | Khnang Sampov | Lower peak | KSP01 | 12 August 2020 |
| KS06 | Fulgoroidea | *Symplanella* sp. | F | Low | Khnang Sampov | Lower peak | KSP01 | 12 August 2020 |
| KS07 | Cercopoidea | *Clovia* sp. | M | Low | Khnang Sampov | Lower peak | KSP01 | 12 August 2020 |
| KS08 | Cercopoidea | *Clovia* sp. | F | Low | Khnang Sampov | Lower peak | KSP01 | 12 August 2020 |
| KS09 | Cercopoidea | *Clovia* sp. | F | Low | Khnang Sampov | Lower peak | KSP01 | 12 August 2020 |
| KS10 | Membracoidea | *Stirellus* sp1. | M | High | Khnang Phsar | Third Peak | KSP02 | 13 August 2020 |
| KS11 | Membracoidea | *Stirellus* sp1. | F | High | Khnang Phsar | Third Peak | KSP02 | 13 August 2020 |
| KS12 | Membracoidea | *Stirellus* sp1. | F | High | Khnang Phsar | Third Peak | KSP02 | 13 August 2020 |
| KS13 | Membracoidea | *Stirellus* sp2. | F | High | Khnang Phsar | Third Peak | KSP02 | 13 August 2020 |
| KS14 | Membracoidea | *Stirellus* sp2. | F | High | Khnang Phsar | Third Peak | KSP02 | 13 August 2020 |
| KS15 | Membracoidea | *Hecalus* sp. | F | High | Khnang Phsar | Third Peak | KSP02 | 13 August 2020 |
| KS16 | Membracoidea | *Hecalus* sp. | F | High | Khnang Phsar | Third Peak | KSP02 | 13 August 2020 |
| KS17 | Membracoidea | *Hecalus* sp. | M | High | Khnang Phsar | Third Peak | KSP02 | 13 August 2020 |
| KS18 | Membracoidea | *Hecalus* sp. | M | High | Khnang Phsar | Third Peak | KSP02 | 13 August 2020 |
| KS19 | Membracoidea | *Chanwhania* sp. | F | High | Khnang Phsar | Third Peak | KSP02 | 13 August 2020 |
| KS20 | Membracoidea | *Chanwhania* sp. | F | High | Khnang Phsar | Third Peak | KSP02 | 13 August 2020 |
| KS21 | Membracoidea | *Chanwhania* sp. | M | High | Khnang Phsar | Third Peak | KSP02 | 13 August 2020 |
| KS22 | Membracoidea | *Stirellus* sp1. | F | High | Khnang Phsar | Second Peak | KSP03 | 13 August 2020 |
| KS23 | Membracoidea | *Stirellus* sp1. | F | High | Khnang Phsar | Second Peak | KSP03 | 13 August 2020 |
| KS24 | Membracoidea | *Stirellus* sp1. | M | High | Khnang Phsar | Second Peak | KSP03 | 13 August 2020 |
| KS25 | Membracoidea | *Stirellus* sp2. | M | High | Khnang Phsar | Second Peak | KSP03 | 13 August 2020 |
| KS26 | Membracoidea | *Stirellus* sp2. | F | High | Khnang Phsar | Second Peak | KSP03 | 13 August 2020 |
| KS27 | Membracoidea | Stirellus sp2. | F | High | Khnang Phsar | Second Peak | KSP03 | 13 August 2020 |
| KS28 | Membracoidea | *Hecalus* sp. | F | High | Khnang Phsar | Second Peak | KSP03 | 13 August 2020 |
| KS29 | Membracoidea | *Hecalus* sp. | F | High | Khnang Phsar | Second Peak | KSP03 | 13 August 2020 |
| KS30 | Membracoidea | *Hecalus* sp. | M | High | Khnang Phsar | Second Peak | KSP03 | 13 August 2020 |
| KS31 | Membracoidea | *Chanwhania* sp. | M | High | Khnang Phsar | Second Peak | KSP03 | 13 August 2020 |
| KS32 | Membracoidea | *Chanwhania* sp. | F | High | Khnang Phsar | Second Peak | KSP03 | 13 August 2020 |
| KS33 | Membracoidea | *Chanwhania* sp. | F | High | Khnang Phsar | Second Peak | KSP03 | 13 August 2020 |
| KS34 | Membracoidea | *Anagonalia* sp. | M | High | Khnang Phsar | Second Peak | KSP03 | 13 August 2020 |
| KS35 | Cercopoidea | *Clovia* sp. | F | High | Khnang Phsar | Second Peak | KSP03 | 13 August 2020 |
| KS36 | Cercopoidea | *Clovia* sp. | M | High | Khnang Phsar | Second Peak | KSP03 | 13 August 2020 |
| KS37 | Membracoidea | *Stirellus* sp1. | M | High | Khnang Sampov | First Peak | KSP04 | 14 August 2020 |
| KS38 | Membracoidea | *Stirellus* sp1. | M | High | Khnang Sampov | First Peak | KSP04 | 14 August 2020 |
| KS39 | Membracoidea | *Stirellus* sp2. | M | High | Khnang Sampov | First Peak | KSP04 | 14 August 2020 |
| KS40 | Membracoidea | *Stirellus* sp2. | M | High | Khnang Sampov | First Peak | KSP04 | 14 August 2020 |
| KS41 | Membracoidea | *Stirellus* sp2. | F | High | Khnang Sampov | First Peak | KSP04 | 14 August 2020 |
| KS42 | Membracoidea | *Stirellus* sp2. | F | High | Khnang Sampov | First Peak | KSP04 | 14 August 2020 |
| KS43 | Membracoidea | *Anagonalia* sp. | M | High | Khnang Sampov | First Peak | KSP04 | 14 August 2020 |
| KS44 | Membracoidea | *Anagonalia* sp. | M | High | Khnang Sampov | First Peak | KSP04 | 14 August 2020 |
| KS45 | Membracoidea | *Anagonalia* sp. | F | High | Khnang Sampov | First Peak | KSP04 | 14 August 2020 |
| KS46 | Membracoidea | *Anagonalia* sp. | F | High | Khnang Sampov | First Peak | KSP04 | 14 August 2020 |
| KS47 | Membracoidea | *Mukaria* sp. | M | High | Khnang Sampov | First Peak | KSP04 | 14 August 2020 |
| KS48 | Membracoidea | *Mukaria* sp. | F | High | Khnang Sampov | First Peak | KSP04 | 14 August 2020 |
| KS49 | Fulgoroidea | *Symplanella* sp. | M | High | Khnang Sampov | First Peak | KSP04 | 14 August 2020 |
| KS50 | Fulgoroidea | *Symplanella* sp. | M | High | Khnang Sampov | First Peak | KSP04 | 14 August 2020 |
| KS51 | Fulgoroidea | *Symplanella* sp. | M | High | Khnang Sampov | First Peak | KSP04 | 14 August 2020 |
| KS52 | Fulgoroidea | *Symplanella* sp. | F | High | Khnang Sampov | First Peak | KSP04 | 14 August 2020 |
| KS53 | Cercopoidea | *Clovia* sp. | F | High | Khnang Sampov | First Peak | KSP04 | 14 August 2020 |
| KS54 | Cercopoidea | *Clovia* sp. | F | High | Khnang Sampov | First Peak | KSP04 | 14 August 2020 |
| KS55 | Cercopoidea | *Clovia* sp. | M | High | Khnang Sampov | First Peak | KSP04 | 14 August 2020 |
| KS56 | Cercopoidea | *Clovia* sp. | M | High | Khnang Sampov | First Peak | KSP04 | 14 August 2020 |
| KS57 | Membracoidea | *Stirellus* sp1. | M | Low | Khnang Phsar | Lower peak | KSP05 | 14 August 2020 |
| KS58 | Membracoidea | *Stirellus* sp1. | M | Low | Khnang Phsar | Lower peak | KSP05 | 14 August 2020 |
| KS59 | Membracoidea | *Stirellus* sp2. | F | Low | Khnang Phsar | Lower peak | KSP05 | 14 August 2020 |
| KS60 | Membracoidea | *Stirellus* sp2. | F | Low | Khnang Phsar | Lower peak | KSP05 | 14 August 2020 |
| KS61 | Membracoidea | *Stirellus* sp2. | M | Low | Khnang Phsar | Lower peak | KSP05 | 14 August 2020 |
| KS62 | Membracoidea | *Stirellus* sp2. | M | Low | Khnang Phsar | Lower peak | KSP05 | 14 August 2020 |
| KS63 | Membracoidea | *Hecalus* sp. | F | Low | Khnang Phsar | Lower peak | KSP05 | 14 August 2020 |
| KS64 | Membracoidea | *Hecalus* sp. | F | Low | Khnang Phsar | Lower peak | KSP05 | 14 August 2020 |
| KS65 | Membracoidea | *Hecalus* sp. | M | Low | Khnang Phsar | Lower peak | KSP05 | 14 August 2020 |
| KS66 | Membracoidea | *Hecalus* sp. | M | Low | Khnang Phsar | Lower peak | KSP05 | 14 August 2020 |
| KS67 | Membracoidea | *Chanwhania* sp. | F | Low | Khnang Phsar | Lower peak | KSP05 | 14 August 2020 |
| KS68 | Membracoidea | *Chanwhania* sp. | F | Low | Khnang Phsar | Lower peak | KSP05 | 14 August 2020 |
| KS70 | Membracoidea | *Chanwhania* sp. | M | Low | Khnang Phsar | Lower peak | KSP05 | 14 August 2020 |
| KS71 | Membracoidea | *Anagonalia* sp. | M | Low | Khnang Phsar | Lower peak | KSP05 | 14 August 2020 |
| KS72 | Membracoidea | *Anagonalia* sp. | M | Low | Khnang Phsar | Lower peak | KSP05 | 14 August 2020 |
| KS73 | Membracoidea | *Anagonalia* sp. | F | Low | Khnang Phsar | Lower peak | KSP05 | 14 August 2020 |
| KS74 | Membracoidea | *Anagonalia* sp. | F | Low | Khnang Phsar | Lower peak | KSP05 | 14 August 2020 |
| KS75 | Membracoidea | *Mukaria* sp. | M | Low | Khnang Phsar | Lower peak | KSP05 | 14 August 2020 |
| KS76 | Membracoidea | *Mukaria* sp. | F | Low | Khnang Phsar | Lower peak | KSP05 | 14 August 2020 |
| KS77 | Membracoidea | *Mukaria* sp. | M | Low | Khnang Phsar | Lower peak | KSP05 | 14 August 2020 |
| KS78 | Membracoidea | *Mukaria* sp. | F | Low | Khnang Phsar | Lower peak | KSP05 | 14 August 2020 |
| KS79 | Fulgoroidea | *Symplanella* sp. | M | Low | Khnang Phsar | Lower peak | KSP05 | 14 August 2020 |
| KS80 | Fulgoroidea | *Symplanella* sp. | F | Low | Khnang Phsar | Lower peak | KSP05 | 14 August 2020 |
| KS81 | Fulgoroidea | *Symplanella* sp. | F | Low | Khnang Phsar | Lower peak | KSP05 | 14 August 2020 |
| KS82 | Cercopoidea | *Clovia* sp. | M | Low | Khnang Phsar | Lower peak | KSP05 | 14 August 2020 |
| KS83 | Cercopoidea | *Clovia* sp. | F | Low | Khnang Phsar | Lower peak | KSP05 | 14 August 2020 |
| KS84 | Cercopoidea | *Clovia* sp. | M | High | Khnang Sampov | Third Peak | KSP02 | 13 August 2020 |

**Note:** Total of 83 Auchenorrhyncha samples were valid in the study. One sample **KS69** was removed from the dataset due to their invalid sequence read from the sequencing facility.

**Table S2**: Read count tracking of sequence reads by sample of the Illumina Miseq (V3-V4) amplicons

| **Sam.ID** | **Host Species** | **dada2 input** | **filtered** | **dada_F** | **dada_R** | **Merged** | **Nonchim** | **Final_reads (%)** |
| --- | --- | --- | --- | --- | --- | --- | --- | --- |
| KS01 | *Stirellus* sp1. | 22866 | 22846 | 19824 | 22589 | 19307 | 16864 | 73.8 |
| KS02 | *Stirellus* sp1. | 5113 | 5107 | 4304 | 5066 | 4204 | 3753 | 73.4 |
| KS03 | *Stirellus* sp1. | 32855 | 32832 | 27346 | 32364 | 26462 | 23452 | 71.4 |
| KS04 | *Symplanella* sp. | 2113 | 2110 | 1989 | 2109 | 1955 | 1826 | 86.4 |
| KS05 | *Symplanella* sp. | 38024 | 38006 | 36217 | 37960 | 35822 | 33388 | 87.8 |
| KS06 | *Symplanella* sp. | 22082 | 22057 | 20455 | 22033 | 20395 | 19707 | 89.2 |
| KS07 | *Clovia* sp. | 4641 | 4638 | 4219 | 4621 | 4196 | 3772 | 81.3 |
| KS08 | *Clovia* sp. | 24179 | 24161 | 22566 | 24108 | 22279 | 20379 | 84.3 |
| KS09 | *Clovia* sp. | 27822 | 27795 | 26220 | 27690 | 25654 | 22753 | 81.8 |
| KS10 | *Stirellus* sp1. | 6770 | 6765 | 5532 | 6689 | 5399 | 4840 | 71.5 |
| KS11 | *Stirellus* sp1. | 8243 | 8235 | 6763 | 8130 | 6565 | 6065 | 73.6 |
| KS12 | *Stirellus* sp1. | 36776 | 36742 | 30383 | 36264 | 29416 | 26586 | 72.3 |
| KS13 | *Stirellus* sp2. | 10943 | 10931 | 9822 | 10711 | 9557 | 8873 | 81.1 |
| KS14 | *Stirellus* sp2. | 19378 | 19370 | 17657 | 19096 | 17288 | 16119 | 83.2 |
| KS15 | *Hecalus* sp. | 12483 | 12473 | 11678 | 12367 | 11364 | 8926 | 71.5 |
| KS16 | *Hecalus* sp. | 15258 | 15238 | 14592 | 15142 | 14171 | 12724 | 83.4 |
| KS17 | *Hecalus* sp. | 24559 | 24526 | 23902 | 24410 | 23484 | 20885 | 85 |
| KS18 | *Hecalus* sp. | 7837 | 7831 | 7667 | 7814 | 7545 | 6919 | 88.3 |
| KS19 | *Chanwhania* sp. | 35123 | 35089 | 32378 | 34946 | 31128 | 28014 | 79.8 |
| KS20 | *Chanwhania* sp. | 32817 | 32779 | 29302 | 32592 | 28087 | 25223 | 76.9 |
| KS21 | *Chanwhania* sp. | 14762 | 14752 | 13418 | 14503 | 12806 | 10272 | 69.6 |
| KS22 | *Stirellus* sp1. | 24619 | 24589 | 22599 | 24489 | 22120 | 21140 | 85.9 |
| KS23 | *Stirellus* sp1. | 5546 | 5544 | 4561 | 5511 | 4427 | 4058 | 73.2 |
| KS24 | *Stirellus* sp1. | 8889 | 8882 | 7340 | 8625 | 7036 | 5917 | 66.6 |
| KS25 | *Stirellus* sp2. | 13852 | 13847 | 13627 | 13629 | 13360 | 12031 | 86.9 |
| KS26 | *Stirellus* sp2. | 36807 | 36769 | 36521 | 36527 | 36014 | 32479 | 88.2 |
| KS27 | Stirellus sp2. | 3656 | 3651 | 3627 | 3632 | 3515 | 3337 | 91.3 |
| KS28 | *Hecalus* sp. | 67086 | 67019 | 63297 | 66603 | 61126 | 54839 | 81.7 |
| KS29 | *Hecalus* sp. | 13190 | 13175 | 12465 | 13131 | 12059 | 10931 | 82.9 |
| KS30 | *Hecalus* sp. | 26995 | 26975 | 25941 | 26839 | 25537 | 24350 | 90.2 |
| KS31 | *Chanwhania* sp. | 10524 | 10516 | 9996 | 10421 | 9662 | 8446 | 80.3 |
| KS32 | *Chanwhania* sp. | 22219 | 22185 | 20740 | 21937 | 19996 | 17238 | 77.6 |
| KS33 | *Chanwhania* sp. | 10344 | 10337 | 9426 | 10287 | 9135 | 8331 | 80.5 |
| KS34 | *Anagonalia* sp. | 23601 | 23575 | 19568 | 23479 | 19449 | 18170 | 77 |
| KS35 | *Clovia* sp. | 18140 | 18126 | 17053 | 18077 | 16598 | 15084 | 83.2 |
| KS36 | *Clovia* sp. | 8459 | 8447 | 7823 | 8416 | 7627 | 6911 | 81.7 |
| KS37 | *Stirellus* sp1. | 1963 | 1963 | 1653 | 1924 | 1577 | 1336 | 68.1 |
| KS38 | *Stirellus* sp1. | 19171 | 19149 | 16820 | 18977 | 16449 | 15084 | 78.7 |
| KS39 | *Stirellus* sp2. | 5497 | 5493 | 5466 | 5467 | 5419 | 5098 | 92.7 |
| KS40 | *Stirellus* sp2. | 29020 | 28991 | 28284 | 28372 | 27456 | 22309 | 76.9 |
| KS41 | *Stirellus* sp2. | 31669 | 31635 | 28276 | 31417 | 27752 | 26597 | 84 |
| KS42 | *Stirellus* sp2. | 9697 | 9693 | 8966 | 9596 | 8779 | 8187 | 84.4 |
| KS43 | *Anagonalia* sp. | 18465 | 18450 | 15532 | 18227 | 15308 | 14007 | 75.9 |
| KS44 | *Anagonalia* sp. | 14597 | 14584 | 12106 | 14361 | 11925 | 11046 | 75.7 |
| KS45 | *Anagonalia* sp. | 39144 | 39106 | 32968 | 38922 | 32749 | 31184 | 79.7 |
| KS46 | *Anagonalia* sp. | 31377 | 31343 | 26070 | 31236 | 25966 | 24659 | 78.6 |
| KS47 | *Mukaria* sp. | 2530 | 2525 | 2353 | 2494 | 2304 | 2106 | 83.2 |
| KS48 | *Mukaria* sp. | 15894 | 15878 | 14788 | 15747 | 14516 | 13146 | 82.7 |
| KS49 | *Symplanella* sp. | 7684 | 7678 | 7180 | 7661 | 7108 | 6555 | 85.3 |
| KS50 | *Symplanella* sp. | 4589 | 4586 | 4338 | 4581 | 4302 | 3978 | 86.7 |
| KS51 | *Symplanella* sp. | 10345 | 10336 | 9667 | 10322 | 9642 | 9150 | 88.4 |
| KS52 | *Symplanella* sp. | 45585 | 45545 | 41620 | 45412 | 41244 | 39014 | 85.6 |
| KS53 | *Clovia* sp. | 36533 | 36498 | 34223 | 36352 | 32550 | 30868 | 84.5 |
| KS54 | *Clovia* sp. | 2811 | 2811 | 2627 | 2807 | 2605 | 2395 | 85.2 |
| KS55 | *Clovia* sp. | 21589 | 21571 | 19811 | 21497 | 18817 | 17111 | 79.3 |
| KS56 | *Clovia* sp. | 47727 | 47685 | 44538 | 47254 | 43455 | 37564 | 78.7 |
| KS57 | *Stirellus* sp1. | 7 | 7 | 6 | 7 | 6 | 4 | 57.1 |
| KS58 | *Stirellus* sp1. | 21227 | 21209 | 17911 | 20755 | 16397 | 13305 | 62.7 |
| KS59 | *Stirellus* sp2. | 12297 | 12290 | 12136 | 12203 | 11932 | 10689 | 86.9 |
| KS60 | *Stirellus* sp2. | 19050 | 19035 | 18852 | 18835 | 18433 | 16857 | 88.5 |
| KS61 | *Stirellus* sp2. | 2518 | 2516 | 2454 | 2453 | 2345 | 2033 | 80.7 |
| KS62 | *Stirellus* sp2. | 17514 | 17494 | 17107 | 17178 | 16593 | 14205 | 81.1 |
| KS63 | *Hecalus* sp. | 10962 | 10951 | 10431 | 10891 | 10139 | 9176 | 83.7 |
| KS64 | *Hecalus* sp. | 16917 | 16899 | 15980 | 16787 | 15554 | 14447 | 85.4 |
| KS65 | *Hecalus* sp. | 17693 | 17677 | 16575 | 17203 | 15770 | 13120 | 74.2 |
| KS66 | *Hecalus* sp. | 18809 | 18789 | 18249 | 18690 | 17900 | 16905 | 89.9 |
| KS67 | *Chanwhania* sp. | 9244 | 9228 | 8448 | 9188 | 8142 | 7484 | 81 |
| KS68 | *Chanwhania* sp. | 7869 | 7863 | 7040 | 7745 | 6677 | 5966 | 75.8 |
| KS70 | *Chanwhania* sp. | 14444 | 14432 | 13093 | 14270 | 12641 | 11575 | 80.1 |
| KS71 | *Anagonalia* sp. | 24783 | 24750 | 20655 | 24430 | 20366 | 19128 | 77.2 |
| KS72 | *Anagonalia* sp. | 38844 | 38800 | 33164 | 36949 | 30672 | 20770 | 53.5 |
| KS73 | *Anagonalia* sp. | 5957 | 5948 | 4944 | 5905 | 4853 | 4405 | 73.9 |
| KS74 | *Anagonalia* sp. | 17184 | 17169 | 14314 | 17054 | 14198 | 13116 | 76.3 |
| KS75 | *Mukaria* sp. | 11394 | 11380 | 10714 | 11278 | 10518 | 9445 | 82.9 |
| KS76 | *Mukaria* sp. | 9148 | 9138 | 8668 | 9108 | 8589 | 8052 | 88 |
| KS77 | *Mukaria* sp. | 11108 | 11098 | 10485 | 11036 | 10296 | 9587 | 86.3 |
| KS78 | *Mukaria* sp. | 92307 | 92199 | 85437 | 91815 | 84352 | 77298 | 83.7 |
| KS79 | *Symplanella* sp. | 6533 | 6519 | 6000 | 6490 | 5915 | 5365 | 82.1 |
| KS80 | *Symplanella* sp. | 27310 | 27285 | 25579 | 27093 | 25140 | 22767 | 83.4 |
| KS81 | *Symplanella* sp. | 67033 | 66969 | 62051 | 66836 | 61704 | 58323 | 87 |
| KS82 | *Clovia* sp. | 80702 | 80616 | 75053 | 80527 | 73790 | 67234 | 83.3 |
| KS83 | *Clovia* sp. | 26812 | 26787 | 23786 | 26657 | 23593 | 21340 | 79.6 |
| KS84 | *Clovia* sp. | 29512 | 29476 | 26562 | 29311 | 25228 | 23084 | 78.2 |

**Note:** Total of 83 Auchenorrhyncha samples were valid in the study. One sample **KS69** was removed from the dataset due to their invalid sequence read from the sequencing facility.

**Table S3**: Relative abundance (sequence reads) of bacterial taxa (ASVs) in association with Auchenorrhyncha insects

| **ASVs** | **Phylum** | **Class** | **Order** | **Family** | **Genus** | **ASVs Abundance** |
| --- | --- | --- | --- | --- | --- | --- |
| ASV_1 | Bacteroidota | Bacteroidia | Flavobacteriales | Blattabacteriaceae | Candidatus Karelsucia | 151194 |
| ASV_2 | Bacteroidota | Bacteroidia | Flavobacteriales | Blattabacteriaceae | Candidatus Karelsucia | 119054 |
| ASV_3 | Bacteroidota | Bacteroidia | Flavobacteriales | Blattabacteriaceae | Candidatus Karelsucia | 114846 |
| ASV_4 | Bacteroidota | Bacteroidia | Flavobacteriales | Blattabacteriaceae | Candidatus Karelsucia | 110583 |
| ASV_6 | Bacteroidota | Bacteroidia | Flavobacteriales | Blattabacteriaceae | Candidatus Karelsucia | 82741 |
| ASV_8 | Proteobacteria | Alphaproteobacteria | Rickettsiales | Rickettsiaceae | Rickettsia | 62016 |
| ASV_10 | Bacteroidota | Bacteroidia | Flavobacteriales | Blattabacteriaceae | Candidatus Karelsucia | 53324 |
| ASV_11 | Proteobacteria | Gammaproteobacteria | Burkholderiales | Oxalobacteraceae | Candidatus Zinderia | 49206 |
| ASV_13 | Bacteroidota | Bacteroidia | Flavobacteriales | Blattabacteriaceae | Candidatus Karelsucia | 36402 |
| ASV_14 | Proteobacteria | Gammaproteobacteria | Enterobacterales | Morganellaceae | Candidatus Symbiodolus | 35815 |
| ASV_15 | Proteobacteria | Alphaproteobacteria | Rickettsiales | Rickettsiaceae | Rickettsia | 35654 |
| ASV_16 | Proteobacteria | Alphaproteobacteria | Rickettsiales | Rickettsiaceae | Rickettsia | 33968 |
| ASV_17 | Proteobacteria | Alphaproteobacteria | Rickettsiales | Fokiniaceae | Candidatus Lariskella | 31734 |
| ASV_19 | Proteobacteria | Alphaproteobacteria | Rickettsiales | Anaplasmataceae | Wolbachia | 24448 |
| ASV_20 | Proteobacteria | Gammaproteobacteria | Enterobacterales | Morganellaceae | Arsenophonus | 23618 |
| ASV_21 | Proteobacteria | Alphaproteobacteria | Rickettsiales | Anaplasmataceae | Wolbachia | 12467 |
| ASV_22 | Proteobacteria | Gammaproteobacteria | Enterobacterales | Pectobacteriaceae | Pectobacterium | 12246 |
| ASV_23 | Proteobacteria | Alphaproteobacteria | Rickettsiales | Anaplasmataceae | Wolbachia | 12163 |
| ASV_24 | Proteobacteria | Gammaproteobacteria | Burkholderiales | Oxalobacteraceae | Candidatus Zinderia | 11134 |
| ASV_27 | Proteobacteria | Gammaproteobacteria | Enterobacterales | Pectobacteriaceae | Pectobacterium | 5835 |
| ASV_28 | Proteobacteria | Gammaproteobacteria | Pseudomonadales | Pseudomonadaceae | Pseudomonas | 4686 |
| ASV_29 | Proteobacteria | Gammaproteobacteria | Xanthomonadales | Xanthomonadaceae | Xylella | 4003 |
| ASV_31 | Firmicutes | Bacilli | Entomoplasmatales | Spiroplasmataceae | Spiroplasma | 2970 |
| ASV_32 | Firmicutes | Bacilli | Entomoplasmatales | Spiroplasmataceae | Spiroplasma | 2959 |
| ASV_33 | Proteobacteria | Gammaproteobacteria | Burkholderiales | Oxalobacteraceae | unclassified_Oxalobacteraceae | 1474 |
| ASV_35 | Proteobacteria | Alphaproteobacteria | Rickettsiales | Rickettsiaceae | Rickettsia | 1228 |
| ASV_38 | Proteobacteria | Gammaproteobacteria | Enterobacterales | Yersiniaceae | unclassified_Yersiniaceae | 671 |
| ASV_40 | Actinobacteriota | Actinobacteria | Micrococcales | Microbacteriaceae | unclassified_Microbacteriaceae | 456 |
| ASV_41 | Proteobacteria | Alphaproteobacteria | Rhizobiales | Beijerinckiaceae | Methylobacterium-Methylorubrum | 445 |
| ASV_44 | Proteobacteria | Alphaproteobacteria | Rickettsiales | Fokiniaceae | Candidatus Lariskella | 386 |
| ASV_46 | Proteobacteria | Gammaproteobacteria | Pseudomonadales | Pseudomonadaceae | Pseudomonas | 364 |
| ASV_47 | Bacteroidota | Bacteroidia | Flavobacteriales | Blattabacteriaceae | Candidatus Karelsucia | 345 |
| ASV_48 | Bacteroidota | Bacteroidia | Flavobacteriales | Blattabacteriaceae | Candidatus Karelsucia | 335 |
| ASV_50 | Bacteroidota | Bacteroidia | Flavobacteriales | Blattabacteriaceae | Candidatus Karelsucia | 319 |
| ASV_52 | Proteobacteria | Gammaproteobacteria | Pseudomonadales | Pseudomonadaceae | Pseudomonas | 302 |
| ASV_53 | Proteobacteria | Gammaproteobacteria | Enterobacterales | Pectobacteriaceae | Pectobacterium | 301 |
| ASV_54 | Proteobacteria | Alphaproteobacteria | Rhizobiales | Beijerinckiaceae | Methylobacterium-Methylorubrum | 297 |
| ASV_55 | Proteobacteria | Gammaproteobacteria | Enterobacterales | Enterobacteriaceae | unclassified_Enterobacteriaceae | 293 |
| ASV_34 | Proteobacteria | Alphaproteobacteria | Rhizobiales | Xanthobacteraceae | unclassified_Xanthobacteraceae | 275 |
| ASV_60 | Proteobacteria | Gammaproteobacteria | Burkholderiales | Comamonadaceae | unclassified_Comamonadaceae | 272 |
| ASV_64 | Bacteroidota | Bacteroidia | Flavobacteriales | Blattabacteriaceae | Candidatus Karelsucia | 242 |
| ASV_66 | Bacteroidota | Bacteroidia | Flavobacteriales | Blattabacteriaceae | Candidatus Karelsucia | 237 |
| ASV_67 | Bacteroidota | Bacteroidia | Flavobacteriales | Blattabacteriaceae | Candidatus Karelsucia | 231 |
| ASV_68 | Proteobacteria | Gammaproteobacteria | Pseudomonadales | Moraxellaceae | Acinetobacter | 229 |
| ASV_69 | Proteobacteria | Gammaproteobacteria | Pseudomonadales | Pseudomonadaceae | Pseudomonas | 226 |
| ASV_72 | Actinobacteriota | Actinobacteria | Micrococcales | Microbacteriaceae | unclassified_Microbacteriaceae | 210 |
| ASV_73 | Actinobacteriota | Actinobacteria | Micrococcales | Microbacteriaceae | Curtobacterium | 210 |
| ASV_78 | Proteobacteria | Alphaproteobacteria | Rhizobiales | Beijerinckiaceae | Methylobacterium-Methylorubrum | 174 |
| ASV_80 | Proteobacteria | Gammaproteobacteria | Pseudomonadales | Moraxellaceae | Acinetobacter | 168 |
| ASV_82 | Proteobacteria | Gammaproteobacteria | Pseudomonadales | Moraxellaceae | Acinetobacter | 164 |
| ASV_83 | Bacteroidota | Bacteroidia | Flavobacteriales | Blattabacteriaceae | Candidatus Karelsucia | 164 |
| ASV_85 | Proteobacteria | Alphaproteobacteria | Rhizobiales | Beijerinckiaceae | 1174-901-12 | 157 |
| ASV_90 | Proteobacteria | Gammaproteobacteria | Pseudomonadales | Moraxellaceae | Acinetobacter | 150 |
| ASV_91 | Proteobacteria | Gammaproteobacteria | Enterobacterales | Enterobacteriaceae | unclassified_Enterobacteriaceae | 147 |
| ASV_92 | Proteobacteria | Gammaproteobacteria | Pseudomonadales | Pseudomonadaceae | unclassified_Pseudomonadaceae | 146 |
| ASV_37 | Proteobacteria | Alphaproteobacteria | Rhizobiales | Rhizobiales Incertae Sedis | Phreatobacter | 144 |
| ASV_93 | Proteobacteria | Alphaproteobacteria | Rhizobiales | Beijerinckiaceae | Methylobacterium-Methylorubrum | 144 |
| ASV_94 | Proteobacteria | Gammaproteobacteria | Pseudomonadales | Moraxellaceae | Acinetobacter | 137 |
| ASV_39 | Proteobacteria | Alphaproteobacteria | Sphingomonadales | Sphingomonadaceae | Sphingomonas | 134 |
| ASV_95 | Bacteroidota | Bacteroidia | Cytophagales | Hymenobacteraceae | Hymenobacter | 134 |
| ASV_96 | Firmicutes | Bacilli | Staphylococcales | Staphylococcaceae | Staphylococcus | 132 |
| ASV_98 | Proteobacteria | Gammaproteobacteria | Pseudomonadales | Moraxellaceae | Acinetobacter | 128 |
| ASV_99 | Firmicutes | Bacilli | Paenibacillales | Paenibacillaceae | Paenibacillus | 124 |
| ASV_100 | Proteobacteria | Gammaproteobacteria | Pseudomonadales | Moraxellaceae | Acinetobacter | 123 |
| ASV_102 | Proteobacteria | Alphaproteobacteria | Rhizobiales | Beijerinckiaceae | 1174-901-12 | 120 |
| ASV_105 | Proteobacteria | Gammaproteobacteria | Pseudomonadales | Moraxellaceae | Acinetobacter | 115 |
| ASV_107 | Proteobacteria | Alphaproteobacteria | Rhizobiales | Rhizobiaceae | Aureimonas | 112 |
| ASV_108 | Firmicutes | Bacilli | Lactobacillales | Vagococcaceae | Vagococcus | 110 |
| ASV_110 | Proteobacteria | Gammaproteobacteria | Enterobacterales | Erwiniaceae | unclassified_Erwiniaceae | 106 |
| ASV_51 | Actinobacteriota | Actinobacteria | Propionibacteriales | Propionibacteriaceae | Cutibacterium | 106 |
| ASV_114 | Proteobacteria | Alphaproteobacteria | Rhizobiales | Beijerinckiaceae | Methylobacterium-Methylorubrum | 99 |
| ASV_115 | Bacteroidota | Bacteroidia | Flavobacteriales | Weeksellaceae | Empedobacter | 99 |
| ASV_88 | Proteobacteria | Alphaproteobacteria | Rhodobacterales | Rhodobacteraceae | unclassified_Rhodobacteraceae | 98 |
| ASV_125 | Proteobacteria | Gammaproteobacteria | Pseudomonadales | Moraxellaceae | Enhydrobacter | 90 |
| ASV_129 | Actinobacteriota | Actinobacteria | Corynebacteriales | Corynebacteriaceae | Corynebacterium | 86 |
| ASV_132 | Proteobacteria | Gammaproteobacteria | Pseudomonadales | Moraxellaceae | Acinetobacter | 80 |
| ASV_135 | Proteobacteria | Alphaproteobacteria | Rhizobiales | Rhizobiaceae | Allorhizobium-Neorhizobium-Pararhizobium-Rhizobium | 79 |
| ASV_42 | Proteobacteria | Alphaproteobacteria | Rhizobiales | Xanthobacteraceae | unclassified_Xanthobacteraceae | 76 |
| ASV_140 | Proteobacteria | Alphaproteobacteria | Caulobacterales | Caulobacteraceae | Brevundimonas | 74 |
| ASV_143 | Proteobacteria | Gammaproteobacteria | Pseudomonadales | Moraxellaceae | Acinetobacter | 72 |
| ASV_145 | Bacteroidota | Bacteroidia | Flavobacteriales | Weeksellaceae | Chryseobacterium | 72 |
| ASV_146 | Proteobacteria | Gammaproteobacteria | Pseudomonadales | Moraxellaceae | Acinetobacter | 71 |
| ASV_147 | Proteobacteria | Alphaproteobacteria | Sphingomonadales | Sphingomonadaceae | Sphingomonas | 70 |
| ASV_150 | Proteobacteria | Gammaproteobacteria | Enterobacterales | Pectobacteriaceae | unclassified_Pectobacteriaceae | 70 |
| ASV_152 | Proteobacteria | Gammaproteobacteria | Pseudomonadales | Moraxellaceae | Acinetobacter | 68 |
| ASV_155 | Proteobacteria | Alphaproteobacteria | Rhizobiales | Beijerinckiaceae | 1174-901-12 | 66 |
| ASV_156 | Proteobacteria | Gammaproteobacteria | Burkholderiales | Oxalobacteraceae | Massilia | 63 |
| ASV_157 | Proteobacteria | Gammaproteobacteria | Burkholderiales | Comamonadaceae | Comamonas | 63 |
| ASV_160 | Actinobacteriota | Actinobacteria | Corynebacteriales | Nocardiaceae | Williamsia | 62 |
| ASV_124 | Proteobacteria | Gammaproteobacteria | Pseudomonadales | Moraxellaceae | Acinetobacter | 60 |
| ASV_165 | Bacteroidota | Bacteroidia | Flavobacteriales | Blattabacteriaceae | Candidatus Karelsucia | 60 |
| ASV_87 | Proteobacteria | Gammaproteobacteria | Burkholderiales | Comamonadaceae | unclassified_Comamonadaceae | 60 |
| ASV_169 | Proteobacteria | Alphaproteobacteria | Rhizobiales | Beijerinckiaceae | Methylobacterium-Methylorubrum | 57 |
| ASV_170 | Actinobacteriota | Actinobacteria | Micrococcales | Microbacteriaceae | unclassified_Microbacteriaceae | 57 |
| ASV_171 | Firmicutes | Bacilli | Paenibacillales | Paenibacillaceae | Paenibacillus | 56 |
| ASV_172 | Firmicutes | Bacilli | Bacillales | Bacillaceae | Bacillus | 55 |
| ASV_175 | Bacteroidota | Bacteroidia | Flavobacteriales | Blattabacteriaceae | Candidatus Karelsucia | 55 |
| ASV_61 | Proteobacteria | Gammaproteobacteria | Burkholderiales | Comamonadaceae | Pelomonas | 54 |
| ASV_179 | Bacteroidota | Bacteroidia | Flavobacteriales | Blattabacteriaceae | Candidatus Karelsucia | 53 |
| ASV_180 | Proteobacteria | Gammaproteobacteria | Pseudomonadales | Moraxellaceae | Acinetobacter | 52 |
| ASV_183 | Actinobacteriota | Actinobacteria | Corynebacteriales | Nocardiaceae | Rhodococcus | 51 |
| ASV_184 | Proteobacteria | Alphaproteobacteria | Rhizobiales | Beijerinckiaceae | 1174-901-12 | 50 |
| ASV_185 | Proteobacteria | Alphaproteobacteria | Sphingomonadales | Sphingomonadaceae | unclassified_Sphingomonadaceae | 49 |
| ASV_188 | Firmicutes | Bacilli | Lactobacillales | unclassified_Lactobacillales | NA | 49 |
| ASV_189 | Acidobacteriota | Acidobacteriae | Acidobacteriales | Acidobacteriaceae (Subgroup 1) | Terriglobus | 48 |
| ASV_190 | Proteobacteria | Gammaproteobacteria | Enterobacterales | unclassified_Enterobacterales | NA | 48 |
| ASV_193 | Bacteroidota | Bacteroidia | Flavobacteriales | Blattabacteriaceae | Candidatus Karelsucia | 47 |
| ASV_194 | Bacteroidota | Bacteroidia | Flavobacteriales | unclassified_Flavobacteriales | NA | 47 |
| ASV_195 | Proteobacteria | Gammaproteobacteria | Burkholderiales | Oxalobacteraceae | Massilia | 46 |
| ASV_197 | Proteobacteria | Alphaproteobacteria | Rhizobiales | Beijerinckiaceae | unclassified_Beijerinckiaceae | 45 |
| ASV_200 | Proteobacteria | Gammaproteobacteria | Pseudomonadales | Moraxellaceae | Acinetobacter | 43 |
| ASV_201 | Proteobacteria | Alphaproteobacteria | Sphingomonadales | Sphingomonadaceae | Sphingomonas | 43 |
| ASV_203 | Bacteroidota | Bacteroidia | Flavobacteriales | Blattabacteriaceae | Candidatus Karelsucia | 43 |
| ASV_119 | Bacteroidota | Bacteroidia | Chitinophagales | Chitinophagaceae | Asinibacterium | 42 |
| ASV_210 | Firmicutes | Bacilli | Lactobacillales | Aerococcaceae | Aerosphaera | 42 |
| ASV_81 | Proteobacteria | Alphaproteobacteria | Caulobacterales | Caulobacteraceae | unclassified_Caulobacteraceae | 42 |
| ASV_213 | Bacteroidota | Bacteroidia | Flavobacteriales | Blattabacteriaceae | Candidatus Karelsucia | 39 |
| ASV_214 | Proteobacteria | Gammaproteobacteria | Pseudomonadales | Moraxellaceae | Acinetobacter | 39 |
| ASV_215 | Proteobacteria | Gammaproteobacteria | Pseudomonadales | Moraxellaceae | Acinetobacter | 39 |
| ASV_216 | Proteobacteria | Gammaproteobacteria | Pseudomonadales | Moraxellaceae | Acinetobacter | 39 |
| ASV_131 | Proteobacteria | Gammaproteobacteria | Burkholderiales | Oxalobacteraceae | Massilia | 38 |
| ASV_221 | Proteobacteria | Gammaproteobacteria | Pseudomonadales | Moraxellaceae | Acinetobacter | 37 |
| ASV_222 | Bacteroidota | Bacteroidia | Flavobacteriales | Blattabacteriaceae | Candidatus Karelsucia | 37 |
| ASV_224 | Proteobacteria | Alphaproteobacteria | Sphingomonadales | Sphingomonadaceae | unclassified_Sphingomonadaceae | 37 |
| ASV_225 | Firmicutes | Bacilli | Lactobacillales | Streptococcaceae | Streptococcus | 36 |
| ASV_59 | Bacteroidota | Bacteroidia | Sphingobacteriales | env.OPS 17 | NA | 36 |
| ASV_84 | Proteobacteria | Gammaproteobacteria | Burkholderiales | Burkholderiaceae | Ralstonia | 35 |
| ASV_228 | Proteobacteria | Alphaproteobacteria | Rhizobiales | Rhizobiaceae | Aureimonas | 34 |
| ASV_230 | Proteobacteria | Gammaproteobacteria | Pasteurellales | Pasteurellaceae | Haemophilus | 34 |
| ASV_232 | Bacteroidota | Bacteroidia | Flavobacteriales | Weeksellaceae | Cloacibacterium | 33 |
| ASV_104 | Proteobacteria | Alphaproteobacteria | Sphingomonadales | Sphingomonadaceae | Sphingomonas | 32 |
| ASV_234 | Proteobacteria | Gammaproteobacteria | Xanthomonadales | Xanthomonadaceae | Stenotrophomonas | 32 |
| ASV_235 | Actinobacteriota | Actinobacteria | Micrococcales | Micrococcaceae | Micrococcus | 32 |
| ASV_237 | Actinobacteriota | Actinobacteria | Kineosporiales | Kineosporiaceae | Quadrisphaera | 31 |
| ASV_245 | Actinobacteriota | Actinobacteria | Kineosporiales | Kineosporiaceae | Kineococcus | 28 |
| ASV_252 | Bacteroidota | Bacteroidia | Flavobacteriales | Blattabacteriaceae | Candidatus Karelsucia | 26 |
| ASV_256 | Proteobacteria | Alphaproteobacteria | Sphingomonadales | Sphingomonadaceae | unclassified_Sphingomonadaceae | 25 |
| ASV_258 | Actinobacteriota | Actinobacteria | Micrococcales | Intrasporangiaceae | Lapillicoccus | 24 |
| ASV_260 | Bacteroidota | Bacteroidia | Sphingobacteriales | Sphingobacteriaceae | Mucilaginibacter | 23 |
| ASV_263 | Firmicutes | Bacilli | Lactobacillales | Enterococcaceae | Enterococcus | 22 |
| ASV_268 | Proteobacteria | Gammaproteobacteria | Xanthomonadales | Rhodanobacteraceae | Luteibacter | 21 |
| ASV_103 | Proteobacteria | Gammaproteobacteria | Burkholderiales | Comamonadaceae | Curvibacter | 19 |
| ASV_274 | Bacteroidota | Bacteroidia | Flavobacteriales | Weeksellaceae | unclassified_Weeksellaceae | 19 |
| ASV_287 | Proteobacteria | Alphaproteobacteria | Sphingomonadales | Sphingomonadaceae | Altererythrobacter | 16 |
| ASV_288 | Firmicutes | Bacilli | Acholeplasmatales | Acholeplasmataceae | Candidatus Phytoplasma | 16 |
| ASV_292 | Proteobacteria | Alphaproteobacteria | Rhizobiales | Rhizobiaceae | unclassified_Rhizobiaceae | 15 |
| ASV_192 | Proteobacteria | Alphaproteobacteria | Reyranellales | Reyranellaceae | Reyranella | 14 |
| ASV_293 | Proteobacteria | Gammaproteobacteria | Burkholderiales | Nitrosomonadaceae | DSSD61 | 14 |
| ASV_294 | Firmicutes | Bacilli | Lactobacillales | Aerococcaceae | Globicatella | 14 |
| ASV_296 | Proteobacteria | Alphaproteobacteria | Sphingomonadales | Sphingomonadaceae | Novosphingobium | 14 |
| ASV_122 | Cyanobacteria | Vampirivibrionia | Obscuribacterales | Obscuribacteraceae | Candidatus Obscuribacter | 13 |
| ASV_300 | Firmicutes | Bacilli | Exiguobacterales | Exiguobacteraceae | Exiguobacterium | 13 |
| ASV_302 | Proteobacteria | Alphaproteobacteria | Rickettsiales | Fokiniaceae | Candidatus Lariskella | 13 |
| ASV_118 | Cyanobacteria | Vampirivibrionia | Obscuribacterales | Obscuribacteraceae | Candidatus Obscuribacter | 11 |
| ASV_117 | Proteobacteria | Alphaproteobacteria | Rhizobiales | Xanthobacteraceae | Bradyrhizobium | 9 |
| ASV_308 | Verrucomicrobiota | Chlamydiae | Chlamydiales | Parachlamydiaceae | unclassified_Parachlamydiaceae | 8 |
| ASV_339 | Bacteroidota | Bacteroidia | Flavobacteriales | Blattabacteriaceae | Candidatus Karelsucia | 8 |
| ASV_97 | Fusobacteriota | Fusobacteriia | Fusobacteriales | Fusobacteriaceae | Fusobacterium | 8 |
| ASV_223 | Actinobacteriota | Actinobacteria | Actinomycetales | Actinomycetaceae | Actinomyces | 7 |
| ASV_342 | Firmicutes | Clostridia | Clostridiales | Clostridiaceae | Proteiniclasticum | 7 |
| ASV_353 | Firmicutes | Bacilli | Lactobacillales | Lactobacillaceae | Lactobacillus | 7 |
| ASV_218 | Actinobacteriota | Coriobacteriia | Coriobacteriales | Atopobiaceae | Atopobium | 6 |
| ASV_247 | Proteobacteria | Alphaproteobacteria | Rhizobiales | Hyphomicrobiaceae | Hyphomicrobium | 6 |
| ASV_357 | Proteobacteria | Gammaproteobacteria | Burkholderiales | Oxalobacteraceae | Massilia | 6 |
| ASV_360 | Proteobacteria | Gammaproteobacteria | Pseudomonadales | Moraxellaceae | Acinetobacter | 5 |
| ASV_361 | Proteobacteria | Gammaproteobacteria | Pseudomonadales | Moraxellaceae | Acinetobacter | 5 |
| ASV_362 | Bacteroidota | Bacteroidia | Cytophagales | Spirosomaceae | unclassified_Spirosomaceae | 5 |
| ASV_181 | Actinobacteriota | Actinobacteria | Corynebacteriales | Mycobacteriaceae | Mycobacterium | 4 |
| ASV_368 | Bacteroidota | Bacteroidia | Flavobacteriales | Blattabacteriaceae | Candidatus Karelsucia | 4 |
| ASV_130 | Actinobacteriota | Actinobacteria | Corynebacteriales | Corynebacteriaceae | Corynebacterium | 3 |
| ASV_208 | Proteobacteria | Gammaproteobacteria | Gammaproteobacteria Incertae Sedis | Unknown Family_4 | Acidibacter | 3 |
| ASV_372 | Patescibacteria | Saccharimonadia | Saccharimonadales | unclassified_Saccharimonadales | NA | 3 |
| ASV_373 | Bacteroidota | Bacteroidia | Flavobacteriales | Blattabacteriaceae | Candidatus Karelsucia | 3 |
| ASV_378 | Bacteroidota | Bacteroidia | Flavobacteriales | Blattabacteriaceae | Candidatus Uzinura | 3 |
| ASV_127 | Proteobacteria | Gammaproteobacteria | Burkholderiales | Comamonadaceae | Polaromonas | 2 |
| ASV_380 | Bacteroidota | Bacteroidia | Flavobacteriales | Blattabacteriaceae | Candidatus Karelsucia | 2 |
| ASV_381 | Bacteroidota | Bacteroidia | Flavobacteriales | Blattabacteriaceae | Candidatus Karelsucia | 2 |
| ASV_289 | Actinobacteriota | Thermoleophilia | Solirubrobacterales | Solirubrobacteraceae | Patulibacter | 1 |
| ASV_384 | Proteobacteria | Gammaproteobacteria | Pseudomonadales | Moraxellaceae | Acinetobacter | 1 |
| ASV_386 | Proteobacteria | Gammaproteobacteria | Burkholderiales | Oxalobacteraceae | Candidatus Zinderia | 1 |
| ASV_387 | Bacteroidota | Bacteroidia | Flavobacteriales | Blattabacteriaceae | Candidatus Karelsucia | 1 |

| Table S4. Endosymbiont bacteria associated with Auchenorrhyncha species (hosts) | | | | | | | |  |
| --- | --- | --- | --- | --- | --- | --- | --- | --- |
| Auchenorrhyncha insect hosts | | |  |  |  | ***n*** | **Symbionts** | |
| Superfamily | **Family** | **Subfamily** | **Tribe** | **Species** | **NCBI Access. No.^+^** |  |  | |
| Membracoidea | Cicadellidae | Cicadellinae | Cicadellini | *Anagonalia* sp. | *PX924297* | 9 | *Karelsulcia** | |
|  |  | Deltocephalinae | Paralimnini | *Changwhania* sp. | *PX924276* | 9 | *Karelsulcia** | |
|  |  |  | Hecalini | *Hecalus* sp. | *PX925090* | 11 | *Karelsulcia*; Arsenophonus^(1)^; Rickettsia** | |
|  |  |  | Mukariini | *Mukaria* sp. | *PX925093* | 6 | *Karelsulcia** | |
|  |  |  | Stenometopiini | *Stirellus* sp1. | *PX925094* | 13 | *Karelsulcia*; Wolbachia^(7)^; Spiroplasma^(1)^; Xylella^(1)^* | |
|  |  |  |  | *Stirellus* sp2. | *PX925092* | 13 | *Karelsulcia*; Wolbachia^(4)^;*  *Ca. Symbiodolus^(6)^* | |
| Fulgoroidea | Caliscelidae | Ommatidiotinae | Augilini | *Symplanella* sp. | *PX923218* | 10 | *Karelsulcia*; Arsenophonus^(6)^; Wolbachia^(9)^* | |
| Cercopoidea | Aphrophoridae |  |  | *Clovia* sp. | *PX925091* | 12 | *Karelsulcia*; Ca. Lariskella*;*  *Ca. Zinderia*; Rickettsia^(5)^;*  *Pectobacterium^(8)^; Xylella^(2)^* | |
| * Endosymbiont detected in every single sample of the host species  ^+^ Indicated specimen vouchers (Auchenorrhyncha insects) in the COI accession number in the NCBI database  ^(n)^ Number of samples detected of the endosymbiont is based on the presence/absent in the samples/species | | | | | | | |  |

| **Pair** | **Name** | **Barcode** | **F-primer (341F)** | **Barcoded primer** |  | **Pair** | **Name** | **Barcode** | **R-primer (805R)** | **Barcoded primer** |
| --- | --- | --- | --- | --- | --- | --- | --- | --- | --- | --- |
| 1 | IllB_1_L_341F_BA | TATCACG | CCTACGGGNGGCWGCAG | TATCACGCCTACGGGNGGCWGCAG |  | 1 | IllA_99_L_805R_BA | ACTGAGA | GACTACHVGGGTATCTAATCC | ACTGAGAGACTACHVGGGTATCTAATCC |
| 2 | IllB_10_L_341F_BA | TACGTCA | CCTACGGGNGGCWGCAG | TACGTCACCTACGGGNGGCWGCAG |  | 2 | IllA_98_L_805R_BA | ACTGACT | GACTACHVGGGTATCTAATCC | ACTGACTGACTACHVGGGTATCTAATCC |
| 3 | IllB_101_L_341F_BA | ACATCAG | CCTACGGGNGGCWGCAG | ACATCAGCCTACGGGNGGCWGCAG |  | 3 | IllA_97_L_805R_BA | ACTGATC | GACTACHVGGGTATCTAATCC | ACTGATCGACTACHVGGGTATCTAATCC |
| 4 | IllB_102_L_341F_BA | ACACTGT | CCTACGGGNGGCWGCAG | ACACTGTCCTACGGGNGGCWGCAG |  | 4 | IllA_96_L_805R_BA | ACTGTGT | GACTACHVGGGTATCTAATCC | ACTGTGTGACTACHVGGGTATCTAATCC |
| 5 | IllB_103_L_341F_BA | ACACACT | CCTACGGGNGGCWGCAG | ACACACTCCTACGGGNGGCWGCAG |  | 5 | IllA_95_L_805R_BA | ACTCGAT | GACTACHVGGGTATCTAATCC | ACTCGATGACTACHVGGGTATCTAATCC |
| 6 | IllB_104_L_341F_BA | ACACAGA | CCTACGGGNGGCWGCAG | ACACAGACCTACGGGNGGCWGCAG |  | 6 | IllA_94_L_805R_BA | ACTCAGT | GACTACHVGGGTATCTAATCC | ACTCAGTGACTACHVGGGTATCTAATCC |
| 7 | IllB_107_L_341F_BA | ACGATGT | CCTACGGGNGGCWGCAG | ACGATGTCCTACGGGNGGCWGCAG |  | 7 | IllA_93_L_805R_BA | ACTCTGA | GACTACHVGGGTATCTAATCC | ACTCTGAGACTACHVGGGTATCTAATCC |
| 8 | IllB_108_L_341F_BA | ACGAGTA | CCTACGGGNGGCWGCAG | ACGAGTACCTACGGGNGGCWGCAG |  | 8 | IllA_92_L_805R_BA | ACTCTCT | GACTACHVGGGTATCTAATCC | ACTCTCTGACTACHVGGGTATCTAATCC |
| 9 | IllB_109_L_341F_BA | ACGAGAT | CCTACGGGNGGCWGCAG | ACGAGATCCTACGGGNGGCWGCAG |  | 9 | IllA_91_L_805R_BA | ACTCTAC | GACTACHVGGGTATCTAATCC | ACTCTACGACTACHVGGGTATCTAATCC |
| 10 | IllB_11_L_341F_BA | TACGAGT | CCTACGGGNGGCWGCAG | TACGAGTCCTACGGGNGGCWGCAG |  | 10 | IllA_9_L_805R_BA | TACTGCA | GACTACHVGGGTATCTAATCC | TACTGCAGACTACHVGGGTATCTAATCC |
| 11 | IllB_110_L_341F_BA | ACGCTAT | CCTACGGGNGGCWGCAG | ACGCTATCCTACGGGNGGCWGCAG |  | 11 | IllA_88_L_805R_BA | ACTACGT | GACTACHVGGGTATCTAATCC | ACTACGTGACTACHVGGGTATCTAATCC |
| 12 | IllB_112_L_341F_BA | AGTCTCA | CCTACGGGNGGCWGCAG | AGTCTCACCTACGGGNGGCWGCAG |  | 12 | IllA_87_L_805R_BA | ACTACAC | GACTACHVGGGTATCTAATCC | ACTACACGACTACHVGGGTATCTAATCC |
| 13 | IllB_114_L_341F_BA | AGTCACT | CCTACGGGNGGCWGCAG | AGTCACTCCTACGGGNGGCWGCAG |  | 13 | IllA_86_L_805R_BA | ACTATGC | GACTACHVGGGTATCTAATCC | ACTATGCGACTACHVGGGTATCTAATCC |
| 14 | IllB_115_L_341F_BA | AGTCAGA | CCTACGGGNGGCWGCAG | AGTCAGACCTACGGGNGGCWGCAG |  | 14 | IllA_85_L_805R_BA | ATGCGAT | GACTACHVGGGTATCTAATCC | ATGCGATGACTACHVGGGTATCTAATCC |
| 15 | IllB_116_L_341F_BA | AGTGAGT | CCTACGGGNGGCWGCAG | AGTGAGTCCTACGGGNGGCWGCAG |  | 15 | IllA_84_L_805R_BA | ATGCACT | GACTACHVGGGTATCTAATCC | ATGCACTGACTACHVGGGTATCTAATCC |
| 16 | IllB_118_L_341F_BA | AGATGCT | CCTACGGGNGGCWGCAG | AGATGCTCCTACGGGNGGCWGCAG |  | 16 | IllA_83_L_805R_BA | ATGCTGT | GACTACHVGGGTATCTAATCC | ATGCTGTGACTACHVGGGTATCTAATCC |
| 17 | IllB_12_L_341F_BA | TACGCTA | CCTACGGGNGGCWGCAG | TACGCTACCTACGGGNGGCWGCAG |  | 17 | IllA_82_L_805R_BA | ATGAGCA | GACTACHVGGGTATCTAATCC | ATGAGCAGACTACHVGGGTATCTAATCC |
| 18 | IllB_120_L_341F_BA | AGACTCT | CCTACGGGNGGCWGCAG | AGACTCTCCTACGGGNGGCWGCAG |  | 18 | IllA_81_L_805R_BA | ATGAGTC | GACTACHVGGGTATCTAATCC | ATGAGTCGACTACHVGGGTATCTAATCC |
| 19 | IllB_121_L_341F_BA | AGACTGA | CCTACGGGNGGCWGCAG | AGACTGACCTACGGGNGGCWGCAG |  | 19 | IllA_80_L_805R_BA | ATGTGCT | GACTACHVGGGTATCTAATCC | ATGTGCTGACTACHVGGGTATCTAATCC |
| 20 | IllB_122_L_341F_BA | AGACACA | CCTACGGGNGGCWGCAG | AGACACACCTACGGGNGGCWGCAG |  | 20 | IllA_8_L_805R_BA | TACTGAC | GACTACHVGGGTATCTAATCC | TACTGACGACTACHVGGGTATCTAATCC |
| 21 | IllB_123_L_341F_BA | AGACGAT | CCTACGGGNGGCWGCAG | AGACGATCCTACGGGNGGCWGCAG |  | 21 | IllA_79_L_805R_BA | ATGTCGT | GACTACHVGGGTATCTAATCC | ATGTCGTGACTACHVGGGTATCTAATCC |
| 22 | IllB_124_L_341F_BA | AGAGTCA | CCTACGGGNGGCWGCAG | AGAGTCACCTACGGGNGGCWGCAG |  | 22 | IllA_78_L_805R_BA | ATCGCAT | GACTACHVGGGTATCTAATCC | ATCGCATGACTACHVGGGTATCTAATCC |
| 23 | IllB_125_L_341F_BA | AGAGATG | CCTACGGGNGGCWGCAG | AGAGATGCCTACGGGNGGCWGCAG |  | 23 | IllA_77_L_805R_BA | ATCGACT | GACTACHVGGGTATCTAATCC | ATCGACTGACTACHVGGGTATCTAATCC |
| 24 | IllB_126_L_341F_BA | AGAGACT | CCTACGGGNGGCWGCAG | AGAGACTCCTACGGGNGGCWGCAG |  | 24 | IllA_76_L_805R_BA | ATCGTGT | GACTACHVGGGTATCTAATCC | ATCGTGTGACTACHVGGGTATCTAATCC |
| 25 | IllB_129_L_341F_BA | AGCATGT | CCTACGGGNGGCWGCAG | AGCATGTCCTACGGGNGGCWGCAG |  | 25 | IllA_74_L_805R_BA | ATCAGAC | GACTACHVGGGTATCTAATCC | ATCAGACGACTACHVGGGTATCTAATCC |
| 26 | IllB_13_L_341F_BA | TAGTCAG | CCTACGGGNGGCWGCAG | TAGTCAGCCTACGGGNGGCWGCAG |  | 26 | IllA_72_L_805R_BA | ATCACTC | GACTACHVGGGTATCTAATCC | ATCACTCGACTACHVGGGTATCTAATCC |
| 27 | IllB_130_L_341F_BA | AGCAGAT | CCTACGGGNGGCWGCAG | AGCAGATCCTACGGGNGGCWGCAG |  | 27 | IllA_71_L_805R_BA | ATCATGC | GACTACHVGGGTATCTAATCC | ATCATGCGACTACHVGGGTATCTAATCC |
| 28 | IllB_131_L_341F_BA | GTATCAC | CCTACGGGNGGCWGCAG | GTATCACCCTACGGGNGGCWGCAG |  | 28 | IllA_70_L_805R_BA | ATCTCAC | GACTACHVGGGTATCTAATCC | ATCTCACGACTACHVGGGTATCTAATCC |
| 29 | IllB_138_L_341F_BA | GTCTACT | CCTACGGGNGGCWGCAG | GTCTACTCCTACGGGNGGCWGCAG |  | 29 | IllA_7_L_805R_BA | TACTCGA | GACTACHVGGGTATCTAATCC | TACTCGAGACTACHVGGGTATCTAATCC |
| 30 | IllB_139_L_341F_BA | GTCTCAT | CCTACGGGNGGCWGCAG | GTCTCATCCTACGGGNGGCWGCAG |  | 30 | IllA_69_L_805R_BA | ATAGCGT | GACTACHVGGGTATCTAATCC | ATAGCGTGACTACHVGGGTATCTAATCC |
| 31 | IllB_14_L_341F_BA | TAGACTG | CCTACGGGNGGCWGCAG | TAGACTGCCTACGGGNGGCWGCAG |  | 31 | IllA_68_L_805R_BA | ATACGCT | GACTACHVGGGTATCTAATCC | ATACGCTGACTACHVGGGTATCTAATCC |
| 32 | IllB_140_L_341F_BA | GTCATAG | CCTACGGGNGGCWGCAG | GTCATAGCCTACGGGNGGCWGCAG |  | 32 | IllA_67_L_805R_BA | ATACTGC | GACTACHVGGGTATCTAATCC | ATACTGCGACTACHVGGGTATCTAATCC |
| 33 | IllB_141_L_341F_BA | GTCATCT | CCTACGGGNGGCWGCAG | GTCATCTCCTACGGGNGGCWGCAG |  | 33 | IllA_66_L_805R_BA | TGCGATA | GACTACHVGGGTATCTAATCC | TGCGATAGACTACHVGGGTATCTAATCC |
| 34 | IllB_143_L_341F_BA | GTCACTA | CCTACGGGNGGCWGCAG | GTCACTACCTACGGGNGGCWGCAG |  | 34 | IllA_63_L_805R_BA | TGCTGTA | GACTACHVGGGTATCTAATCC | TGCTGTAGACTACHVGGGTATCTAATCC |
| 35 | IllB_147_L_341F_BA | GATACTG | CCTACGGGNGGCWGCAG | GATACTGCCTACGGGNGGCWGCAG |  | 35 | IllA_62_L_805R_BA | TGCTAGA | GACTACHVGGGTATCTAATCC | TGCTAGAGACTACHVGGGTATCTAATCC |
| 36 | IllB_15_L_341F_BA | TAGACGA | CCTACGGGNGGCWGCAG | TAGACGACCTACGGGNGGCWGCAG |  | 36 | IllA_61_L_805R_BA | TGAGCAT | GACTACHVGGGTATCTAATCC | TGAGCATGACTACHVGGGTATCTAATCC |
| 37 | IllB_152_L_341F_BA | GATGAGT | CCTACGGGNGGCWGCAG | GATGAGTCCTACGGGNGGCWGCAG |  | 37 | IllA_60_L_805R_BA | TGAGTGA | GACTACHVGGGTATCTAATCC | TGAGTGAGACTACHVGGGTATCTAATCC |
| 38 | IllB_153_L_341F_BA | GACTATG | CCTACGGGNGGCWGCAG | GACTATGCCTACGGGNGGCWGCAG |  | 38 | IllA_6_L_805R_BA | TACTCTC | GACTACHVGGGTATCTAATCC | TACTCTCGACTACHVGGGTATCTAATCC |
| 39 | IllB_156_L_341F_BA | GACTCTA | CCTACGGGNGGCWGCAG | GACTCTACCTACGGGNGGCWGCAG |  | 39 | IllA_58_L_805R_BA | TGAGTAC | GACTACHVGGGTATCTAATCC | TGAGTACGACTACHVGGGTATCTAATCC |
| 40 | IllB_157_L_341F_BA | GACTGAT | CCTACGGGNGGCWGCAG | GACTGATCCTACGGGNGGCWGCAG |  | 40 | IllA_57_L_805R_BA | TGACACT | GACTACHVGGGTATCTAATCC | TGACACTGACTACHVGGGTATCTAATCC |
| 41 | IllB_158_L_341F_BA | GACATCA | CCTACGGGNGGCWGCAG | GACATCACCTACGGGNGGCWGCAG |  | 41 | IllA_56_L_805R_BA | TGACTCA | GACTACHVGGGTATCTAATCC | TGACTCAGACTACHVGGGTATCTAATCC |
| 42 | IllB_16_L_341F_BA | TAGAGAG | CCTACGGGNGGCWGCAG | TAGAGAGCCTACGGGNGGCWGCAG | | 42 | IllA_54_L_805R_BA | TGTGCTA | GACTACHVGGGTATCTAATCC | TGTGCTAGACTACHVGGGTATCTAATCC |
| 43 | IllB_160_L_341F_BA | GAGTCAT | CCTACGGGNGGCWGCAG | GAGTCATCCTACGGGNGGCWGCAG |  | 43 | IllA_53_L_805R_BA | TGTGTCA | GACTACHVGGGTATCTAATCC | TGTGTCAGACTACHVGGGTATCTAATCC |
| 44 | IllB_162_L_341F_BA | GAGAGAT | CCTACGGGNGGCWGCAG | GAGAGATCCTACGGGNGGCWGCAG | | 44 | IllA_52_L_805R_BA | TGTCGTA | GACTACHVGGGTATCTAATCC | TGTCGTAGACTACHVGGGTATCTAATCC |
| 45 | IllB_163_L_341F_BA | GCTATCA | CCTACGGGNGGCWGCAG | GCTATCACCTACGGGNGGCWGCAG |  | 45 | IllA_51_L_805R_BA | TGTCACA | GACTACHVGGGTATCTAATCC | TGTCACAGACTACHVGGGTATCTAATCC |
| 46 | IllB_164_L_341F_BA | GCTACTA | CCTACGGGNGGCWGCAG | GCTACTACCTACGGGNGGCWGCAG |  | 46 | IllA_5_L_805R_BA | TACTAGC | GACTACHVGGGTATCTAATCC | TACTAGCGACTACHVGGGTATCTAATCC |
| 47 | IllB_165_L_341F_BA | GCTCATA | CCTACGGGNGGCWGCAG | GCTCATACCTACGGGNGGCWGCAG |  | 47 | IllA_48_L_805R_BA | TCGCATA | GACTACHVGGGTATCTAATCC | TCGCATAGACTACHVGGGTATCTAATCC |
| 48 | IllB_166_L_341F_BA | GCATACT | CCTACGGGNGGCWGCAG | GCATACTCCTACGGGNGGCWGCAG |  | 48 | IllA_44_L_805R_BA | TCGTAGA | GACTACHVGGGTATCTAATCC | TCGTAGAGACTACHVGGGTATCTAATCC |
| 49 | IllB_167_L_341F_BA | GCATCAT | CCTACGGGNGGCWGCAG | GCATCATCCTACGGGNGGCWGCAG |  | 49 | IllA_43_L_805R_BA | TCAGCTA | GACTACHVGGGTATCTAATCC | TCAGCTAGACTACHVGGGTATCTAATCC |
| 50 | IllB_17_L_341F_BA | TAGAGCA | CCTACGGGNGGCWGCAG | TAGAGCACCTACGGGNGGCWGCAG |  | 50 | IllA_42_L_805R_BA | TCAGAGA | GACTACHVGGGTATCTAATCC | TCAGAGAGACTACHVGGGTATCTAATCC |

**Table S5.** Illumina MiSeq Primers (341F/805R) of 50 different barcoding primer pairs

**Table S6.** *Kruskal-Wallis* test and Pairwise comparisons of *Wilcoxon* rank sum test

| ***Observed by species (Obs)*** | | | | | | | | |
| --- | --- | --- | --- | --- | --- | --- | --- | --- |
| *Kruskal-Walis* chi-squared = 28.091, df = 7, *p-*value = *0.0002117* | | | | | | | | |
| *Pairwise comparisons* using *Wilcoxon* rank sum test | | | | | | | | |
|  | *Anagonalia* sp. | *Changwhania* sp. | *Clovia* sp. | *Hecalus* sp. | *Mukaria* sp. | *Stirellus* sp1. | *Stirellus* sp2. |  |
| *Changwhania* sp. | 0.4409 | - | - | - | - | - | - |  |
| *Clovia* sp. | 0.7831 | 0.0731 | - | - | - | - | - |  |
| *Hecalus* sp. | 0.1415 | *0.0082* | 0.0602 | - | - | - | - |  |
| *Mukaria* sp. | 0.8451 | 0.6172 | 0.5989 | 0.0643 | - |  | - |  |
| *Stirellus* sp1. | 0.5782 | 0.8451 | 0.0647 | *0.0081* | 0.5989 | - | - |  |
| *Stirellus* sp2. | 0.8451 | 0.1415 | 0.9347 | 0.0731 | 0.7831 | 0.1415 | - |  |
| *Symplanella* sp. | 0.1415 | *0.0173* | 0.0731 | 0.8451 | 0.0731 | *0.0086* | 0.1160 |  |
|  | | | | | | | | |
| ***Shannon by species (H)*** | | | | | | | | |
| *Kruskal-Walis* chi-squared = 57.384, df = 7, *p-*value = *5.014e-10* | | | | | | | | |
| *Pairwise comparisons* using *Wilcoxon* rank sum test | | | | | | | | |
|  | *Anagonalia* sp. | *Changwhania* sp. | *Clovia* sp. | *Hecalus* sp. | *Mukaria* sp. | *Stirellus* sp1. | *Stirellus* sp2. |  |
| *Changwhania* sp. | 0.08337 | - | - | - | - | - | - |  |
| *Clovia* sp. | *4.8e-05* | *4.8e-05* | *-* | - | - | - | - |  |
| *Hecalus* sp. | *6.7e-05* | *9.5e-05* | *0.00394* |  | - | - | - |  |
| *Mukaria* sp. | 0.73861 | 0.15815 | *0.00090* | *0.00117* | - | - | - |  |
| *Stirellus* sp1. | *0.00753* | 0.39661 | *4.1e-05* | *0.00050* | 0.10290 | - | - |  |
| *Stirellus* sp2. | *0.00417* | *0.02907* | *1.1e-05* | *0.00795* | *0.02064* | 0.11313 | - |  |
| *Symplanella* sp. | *0.00081* | *0.00558* | *9.5e-05* | *0.10290* | *0.00533* | *0.01735* | 0.11313 |  |
|  |  |  |  |  |  |  |  |  |

Note: ‘*Italic’* value is representing a significant difference in comparison between groups of analysis.

**Table S7.** A proportion of host plants composition presented at the sampling sites

| **Sample_site** | **KPS01** | **KPS02** | **KPS03** | **KPS04** | **KPS05** |
| --- | --- | --- | --- | --- | --- |
| Asparagaceae | 0 | 0.02666667 | 0 | 0 | 0 |
| Asteraceae | 0 | 0.03333333 | 0 | 0 | 0 |
| Cyperaceae | 0.01333333 | 0.03333333 | 0 | 0.04 | 0 |
| Dennstaedtiaceae | 0 | 0.00666667 | 0 | 0 | 0 |
| Droseraceae | 0 | 0 | 0 | 0.01 | 0 |
| Eriocaulaceae | 0 | 0.08333333 | 0 | 0 | 0 |
| Fabaceae | 0 | 0.06666667 | 0.01666667 | 0.00666667 | 0 |
| Orchidaceae | 0 | 0.11 | 0 | 0 | 0 |
| Passifloraceae | 0 | 0 | 0 | 0 | 0.00666667 |
| Poaceae | 0.98 | 0.5 | 0.9 | 0.88333333 | 0.96 |
| Proteaceae | 0 | 0.10666667 | 0 | 0.03333333 | 0 |
| Pterichceae | 0 | 0 | 0.01666667 | 0 | 0 |
| Urticaceae | 0 | 0 | 0 | 0 | 0.00666667 |
| Zingiberaceae | 0.00666667 | 0.03333333 | 0.06666667 | 0.02666667 | 0.02666667 |
