# Supplementary material for: Symbiotic Diversity of Sap-Feeding Auchenorrhyncha (Hemiptera) in the Upland Landscapes of Central Cardamom Mountains, Cambodia
Source: Microb Ecol. 2026 Feb 28;89:71. doi: 10.1007/s00248-026-02724-3 (PMC12999766; doi:10.1007/s00248-026-02724-3)
Supplement: Supplementary file 2 [file 248_2026_2724_MOESM2_ESM.docx]

***Symbiotic diversity of sap-feeding Auchenorrhyncha (Hemiptera) in the upland landscapes of Central Cardamom Mountains, Cambodia***

**Sophany Phauk^1,2^*, Sopha Sin^3^ and Olle Terenius^1^**

^1^	Department of Cell and Molecular Biology, Microbiology, Uppsala University, Uppsala, Sweden

^2^	Department of Biology, Faculty of Science, Royal University of Phnom Penh, Cambodia

^3^	Centre for Biodiversity Conservation, Faculty of Science, Royal University of Phnom Penh, Cambodia

*	Corresponding author

Email address: [sophany.phauk@icm.uu.se](mailto:sophany.phauk@icm.uu.se)

**Figure S1.** Positive Control: two samples from the ZymoBIOMICS Microbial Community Standard (Zymo Research) were used as positive controls to evaluate our protocol. When processed along the same pipeline together with the samples from this study, 6 out of 8 bacterial strains were identified correctly at the genus level. Salmonella enterica was classified only down to the Family level as Enterobacteriaceae, while Bacillus subtilis was undetected. Most of the bacteria had proportions close to the expected ones (14%), with Listeria being the main under-represented, and Enterococcus being slightly over-represented. This might indicate a bias in the community composition skewed towards Enterococcacea.

**Figure S2.** Composition of symbiotic bacterial associated with Auchenorrhyncha insects at the genus level. The phylogenetic tree was visualized by using Interactive Tree of Life web portal iTOL vrs. 7.2.1.

**Figure S3.** Composition of symbiotic bacterial associated with Auchenorrhyncha insects at the genus level. The phylogenetic tree was visualized by using Interactive Tree of Life web portal iTOL vrs. 7.2.1.

**Figure S4.** Composition of symbiotic bacterial associated with Auchenorrhyncha insects at the genus level. (A) Bacterial composition at the different exposure level and (B) Comparison of bacterial composition across six species from two different upland landscapes (Khnang Phsar and Khnang Sampov mountain).
